# Sustained blood glutamate scavenging enhances protection in ischemic stroke

**DOI:** 10.1038/s42003-020-01406-1

**Published:** 2020-12-03

**Authors:** Ahlem Zaghmi, Antonio Dopico-López, María Pérez-Mato, Ramón Iglesias-Rey, Pablo Hervella, Andrea A. Greschner, Ana Bugallo-Casal, Andrés da Silva, María Gutiérrez-Fernández, José Castillo, Francisco Campos Pérez, Marc A. Gauthier

**Affiliations:** 1 Institut National de la Recherche Scientifique (INRS), EMT Research Center, Varennes, Qc J3X 1S2 Canada; 2 Clinical Neuroscience Research Laboratory, Health Research Institute of Santiago de Compostela (IDIS), Santiago de Compostela, Spain; 3 Neuroscience and Cerebrovascular Research Laboratory, Department of Neurology and Stroke Center, La Paz University Hospital, Neuroscience Area of IdiPAZ Health Research Institute, Universidad Autónoma de Madrid, Madrid, Spain

**Keywords:** Stroke, Recombinant protein therapy

## Abstract

Stroke is a major cause of morbidity, mortality, and disability. During ischemic stroke, a marked and prolonged rise of glutamate concentration in the brain causes neuronal cell death. This study explores the protective effect of a bioconjugate form of glutamate oxaloacetate transaminase (hrGOT), which catalyzes the depletion of blood glutamate in the bloodstream for ~6 days following a single administration. When treated with this bioconjugate, a significant reduction of the infarct volume and a better retention of sensorimotor function was observed for ischemic rats compared to those treated with saline. Moreover, the equivalent dose of native hrGOT yielded similar results to the saline treated group for some tests. Targeting the bioconjugate to the blood-brain-barrier did not improve its performance. The data suggest that the bioconjugates draw glutamate out of the brain by displacing homeostasis between the different glutamate pools of the body.

## Abstract (web summary)

Using a rodent model of ischemic stroke, Zahgmi et al. report on the neuro-protective effects of a bioconjugate form of the glutamate scavenger, glutamate-oxaloacetate transaminase (GOT). They found that a single dose of the bioconjugate was more effective in reducing the infarct size and improving motor function than the native GOT due to its higher and long lasting blood activity.

## Introduction

Stroke is the second leading cause of death and the third leading cause of disability in the world^1^. According to the World Health Organization, ~5.7 M deaths and 16 M first-ever strokes occurred in 2005 and these numbers may reach 7.8 M and 23 M by 2030, respectively^2^. Current therapeutic approaches for this disease are limited to pharmacological or mechanical recanalization treatments (<15–20% of patients) and unfortunately, even in the best of cases, 80% of stroke patients receive no treatment whatsoever. As a result, <40% of stroke patients have good clinical outcomes. After ischemic stroke, a rapid increase of extracellular glutamate levels results in a permanent influx of calcium and sodium, an over-depolarization of the postsynaptic neuron, and ultimately neuronal death through excitotoxicity^3,4^. Glutamate, the major excitatory neurotransmitter in the brain, is functionally involved in almost all activities of the nervous system but is especially important for learning, memory, and behavior. Unlike other neurotransmitters, the regulation of extracellular glutamate is unique in that there are no extra-synaptic enzymes for its degradation^5^. Instead, homeostasis of the extracellular glutamate concentration is maintained through cellular fast uptake by transporters. Consequently, substantial effort has been devoted to develop protective drugs that either inhibit glutamate receptors (e.g., *N*-methyl-d-aspartate [NMDA] receptor antagonists) or to reduce the increase of calcium ions within neurons (e.g., blockers of voltage sensitive calcium channels). Unfortunately, the administration of such molecules leads to serious clinical side effects. For example, in the case of NMDA receptor antagonists, the lack of discrimination between the diverse actions of the receptor interferes with both the negative and positive aspects of its signaling^4,6^. Furthermore, NMDA antagonists affect glutamate transporters that reside in many extra-cerebral peripheral tissues and play an important role in the metabolic regulation of glutamate^7–9^.

In light of the shortcomings of the current therapeutic approaches and the central role of glutamate in the ischemic cascade, an alternative therapeutic strategy is necessary to address glutamate excitotoxicity. One such approach relies on exploiting the natural diffusion of cerebral glutamate across the blood–brain barrier (BBB) for therapeutic purposes. This is an emerging, conceptually novel protective strategy to reduce the excitotoxic effect of excess extracellular glutamate that accumulates in the brain after ischemic damage. The blood/brain glutamate scavenging mechanism is based on depleting blood glutamate to increase the natural glutamate concentration gradient between the brain and the blood, thereby promoting the efflux of extracellular brain glutamate toward the blood. The main advantage of this strategy is that it involves manipulation of blood chemistry (outside the brain), and therefore does not affect normal brain neurophysiology as happened with drugs antagonists against glutamate receptors. This differs significantly from other drug treatments used to address glutamate excitotoxicity. One of the most efficient pharmacological strategies developed to scavenge blood glutamate consists of the exogenous administration of the blood-resident enzyme, glutamate oxaloacetate transaminase 1 (GOT1, also clinically known as aspartate aminotransferase [AST]). GOT1 has a critical role in the regulation of glutamate levels in blood by catalyzing the reversible transformation of oxaloacetate and glutamate to aspartate and α- ketoglutarate. Thus, the administration of the purified recombinant form of human GOT1 (hrGOT) in ischemic animal models leads to metabolization and reduction of glutamate in the blood and consequently also in the cerebral parenchyma, which is associated with a reduction of the ischemic lesion and better sensorimotor recovery^5,10,11^. However, one major shortcoming of this approach is that the effect of hrGOT administration is short-lived (~3 h), compared with the therapeutic time window required to attenuate glutamate toxicity in brain (~6–9 h after stroke), mainly because of its rapid degradation in the body^12^. In this context, repeated administration or continuous infusion of hrGOT could extend the therapeutic dose of the treatment, though the use of high amounts of a recombinant protein such as hrGOT is not always desirable because of the risk of an immune response. Moreover, although attempts to reduce glutamate levels in the blood are of great importance, the proposed mechanism of protection—glutamate efflux—has not been completely elucidated. Indeed, although it has been shown that high blood GOT levels yield better neurological outcomes, the overexpression of GOT in the brain of ischemic mice has also been shown to reduce the volume of the ischemic stroke lesion, attenuate neurodegeneration, and improve post-stroke sensorimotor function^13^. As such, targeting hrGOT to the brain may have a greater localized effect on the brain glutamate pool, leading to better protection.

This study is the first to evaluate protection of the brain after ischemic stroke using protein bioconjugates with sustained activity in the bloodstream following a single administration. As the transport across the BBB of various drugs or carriers remains a great challenge, the described therapeutic strategy represents a promising paradigm for treating glutamate-brain diseases. Substantially greater protection was achieved using the hrGOT bioconjugates compared with the native hrGOT, in terms of infarct size and retention of motor functions using sensorimotor tests, which are important clinical outputs. Moreover, insight into the mechanism of protection (glutamate efflux) as well as factors for improving the therapy are assessed by examining glutamate levels in the blood and cerebrospinal fluid (CSF), as well as by targeting the hrGOT bioconjugates to the BBB in order to explore a possible localized effect on the brain.

## Results

### Bioconjugate design for sustained blood activity and BBB-targeting

**Fig. 1:**
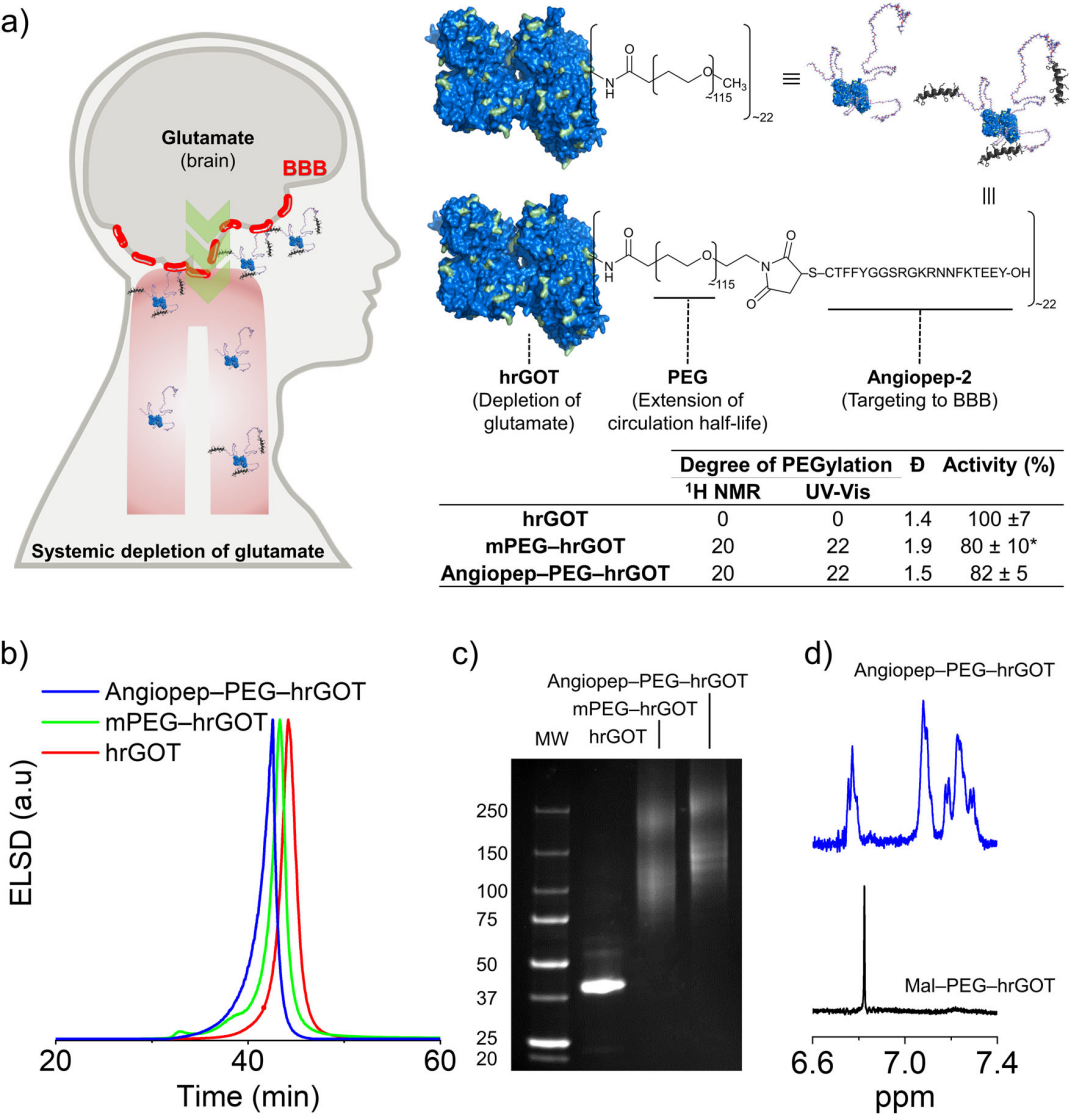
Design and characterization of protective bioconjugates. **a** hrGOT was modified with mPEG or PEG bearing terminal Angiopep-2 ligands, with low loss of catalytic activity. The bioconjugates were designed for extended blood circulation and for BBB accumulation (via Angiopep-2). The degree of PEGylation was determined by UV-Vis and ^1^H NMR spectroscopy. Dispersity was determined by size-exclusion chromatography. Representative **b** size-exclusion chromatograms and **c** SDS–PAGE of the bioconjugates. **d** ^1^H NMR spectra demonstrating quantitative modification of maleimide groups with Angiopep-2. ELSD: evaporative light scattering detector. **a** The star denotes a statistically significant difference with respect to native hrGOT (ANOVA, Tukey, *p* < 0.05). Data presented as mean ± SD, *n* = 3.

HrGOT is a dimeric protein composed of two identical monomers, each with a molecular weight of ~46 kDa^14^. As seen in Fig. 1a, the lysine residues of hrGOT (green) are homogeneously distributed on the surface of the protein and were selected as targets for random modification with mPEG. Dimeric hrGOT possesses 46 pendant amino groups (i.e., 44 lysine residues and 2 N-termini), though only 22 are predicted to be solvent-accessible from the protein’s crystal structure (Table S1). These were quantitatively modified with either amino-reactive mPEG (5 kDa) or α-maleimide, ω-succinimidyl carboxymethyl ester PEG (Mal–PEG–NHS; 5 kDa) as determined by two complementary methods that were in good agreement with one another (Fig. 1a). Thus, the bioconjugates bore ~22 polymer chains and possessed monomodal size-distribution profiles with dispersities (*Đ*) in the range of 1.4–1.9 (Fig. 1b). To target hrGOT to the BBB and potentially promote transport into the brain, the terminal maleimide groups on Mal–PEG–hrGOT were modified with a brain-targeting peptide Angiopep-2, which possesses a single thiol group. Angiopep-2 is a 19-amino acid peptide derived from the common sequence of the low-density lipoprotein receptor-related protein-1 (LRP1) ligands. It is used to target a wide variety of nanocarriers, proteins, or genetic material to the central nervous system^15^. LRP1 is highly-expressed on the luminal side of the BBB and exhibits higher transcytosis efficacy and parenchymal accumulation than other receptors, including those for transferrin, lactoferrin, and avidin^16^. Grafting of Angiopep-2 to the protein bioconjugate was quantitative by ^1^H NMR spectroscopy (via the disappearance of the peak at 6.86 ppm; Fig. 1d) and resulted in an increase in hydrodynamic size as observed by SEC (Fig. 1b). SDS–PAGE analysis of hrGOT bioconjugates under denaturing conditions is shown in Fig. 1c. Two populations of bands were observed for the bioconjugates, representing both the monomeric or dimeric protein (due to incomplete denaturation) modified with polymer chains. No residual unmodified protein was observed, and the size of both bioconjugate populations increased slightly upon modification with Antiopep-2.

**Fig. 2:**
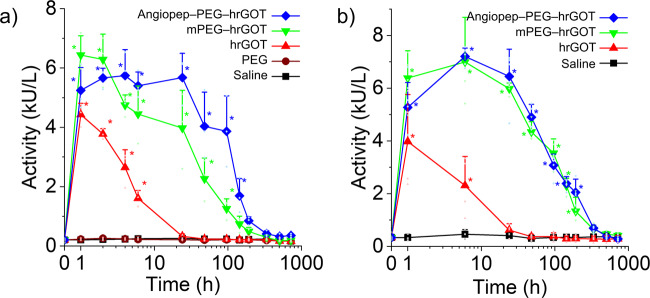
Blood pharmacokinetics. hrGOT activity was measured in **a** healthy and **b** MCAo rats. Stars denote statistically significant differences (ANOVA, Tukey, *p* < 0.05) with respect to the basal GOT activity. Data presented as mean + SD, *n*= 3–5.

GOT catalyzes the reversible reaction of l-aspartate and α-ketoglutarate into oxaloacetate and l-glutamate via a ping–pong mechanism, with pyridoxal 5-phosphate as an essential cofactor^17^. Considering that PEGylation is often associated with loss of catalytic activity^18^, the activity of mPEG–hrGOT and Angiopep–PEG–hrGOT was analyzed by an AST assay. Both bioconjugates possessed ~80% of the activity of the native protein (Fig. 1a). To examine the effect of polymer conjugation on blood exposure, equivalent amounts of native hrGOT, mPEG–hrGOT, or Angiopep–PEG–hrGOT (protein basis, similar activity) were intravenously injected into healthy and ischemic rats. Blood was then withdrawn over a period of 30 days to analyze pharmacokinetics and pharmacodynamics. Note that prior to inducing the ischemic lesion by MCAo, there were no significant differences in endogenous GOT activity between the different treatment groups. As illustrated in Fig. 2a, the initial activity (<1 h) for the hrGOT and bioconjugates groups were similar, in line with the similar enzymatic activity of hrGOT bioconjugates and native hrGOT, as discussed above. Native hrGOT was rapidly cleared from the body of both healthy and MCAo rats, with complete loss of activity in under 6 h. Moreover, no difference was observed between the saline group and the free mPEG group, indicating that mPEG itself has no effect on the enzymatic activity of endogenous GOT. In contrast, the administration of mPEG–hrGOT and Angiopep–PEG–hrGOT maintained high levels of GOT activity between 3–6 kU L^−1^ over a period of ~6 days. This suggests a tentative protection of hrGOT from degradation (by serum proteases), as has been shown for other PEGylated proteins^19,20^. Very little difference, if any, was observed between the mPEG–hrGOT and Angiopep–PEG–hrGOT groups. The pharmacokinetic profiles observed in healthy rats were also very similar to those observed for ischemic rats (Fig. 2b), with negligible differences in half-lives of circulation/elimination or in area under the curves (Table S2). This suggests that routes of distribution and elimination of the bioconjugates were not affected by MCAo and that the extended blood residence time correlated with a delayed elimination half-life.

### Protection

**Fig. 3:**
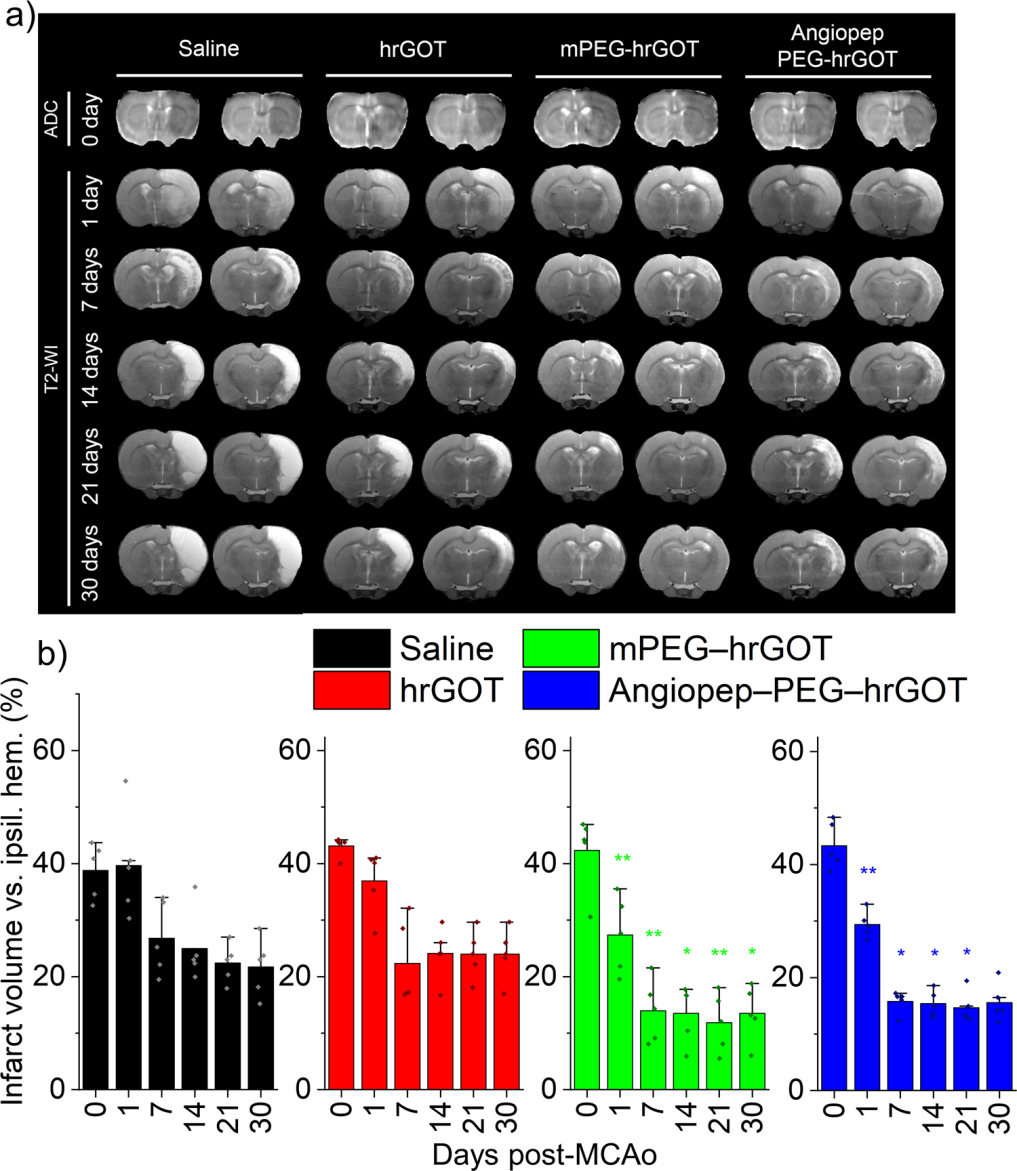
Protective effects assessed by MRI. **a** Representative T_2_W images (slices 7–8 at each per time point) for rats post-MCAo (all other slices are in Table S3). **b** Infarct expressed as percent infarct of total ipsilateral cortex. Stars denote statistically significant differences with respect to the saline group (repeated measures ANOVA, Bonferroni, *p ≤ 0.05, **p ≤ 0.01). Data presented as mean + SD, *n* = 5.

To evaluate the effect of enhanced hrGOT blood exposure imparted by PEGylation, infarct volume was measured by MRI at different times after MCAo. Representative segmented images from a central brain slices for each group are shown in Fig. 3a to illustrate the evolution of infarct area with time. These slices show the occluded territory of the MCA, which is where the main ischemic region is located. The corresponding full stacks of these images are available in Table S3 of the Supporting Information. MRI-based infarct size analysis from the full stacks of images was performed at day 1 and at days 7, 14, 21, and 30 after ischemic inductions and presented in Fig. 3b. MRI data determined using ADC maps confirmed similar baseline lesion volumes between 35% and 45% of the ipsilateral hemisphere in all included animals before treatment administration. T_2_W images were used to measure infarct volume (Fig. 3a). At the dose of hrGOT chosen for these experiments, no statistically significant difference was observed between the hrGOT and saline treated groups, one day after MCAo, in terms of infarct volume. In contrast, treatment with either bioconjugate resulted in a significant ~40% reduction in infarct volume in comparison to saline group (*p* ≤ 0.01). From day 7 onwards, the reduction of the infarct volume remained significant (when compared with saline) and evolved to a lesser extent (for all the groups). When looking closely at the pharmacokinetic curves, one can notice that the enzymatic activity is substantially higher for the bioconjugates than for hrGOT within the first 24 h after ischemia. Knowing that excitotoxicity is a process that starts within the first few mins/hours after ischemia, the initial 24 h are critical for an effective reduction of the cell death. Therefore, maintaining high and sustained enzymatic activity during the initial 24 h is most likely the explanation for the significant impact of the bioconjugates on the size of the infarct at Day 1. Obviously, this does not exclude the fact that maintenance of such activity during the next few days following ischemia continues to provide neuroprotection, and may also contribute to healing processes yet to be identified. Indeed, at Day 30, the infarct volume for the bioconjugate groups was ~30% of its Day 0 value, compared with ~60% for the saline and hrGOT groups, which is comparable to that obtained with hrGOT elsewhere (combined or not with oxaloacetate)^11^. This result thus suggests that sustained GOT activity in the blood, achieved with the bioconjugates, is beneficial for recovery post-MCAo.

**Fig. 4:**
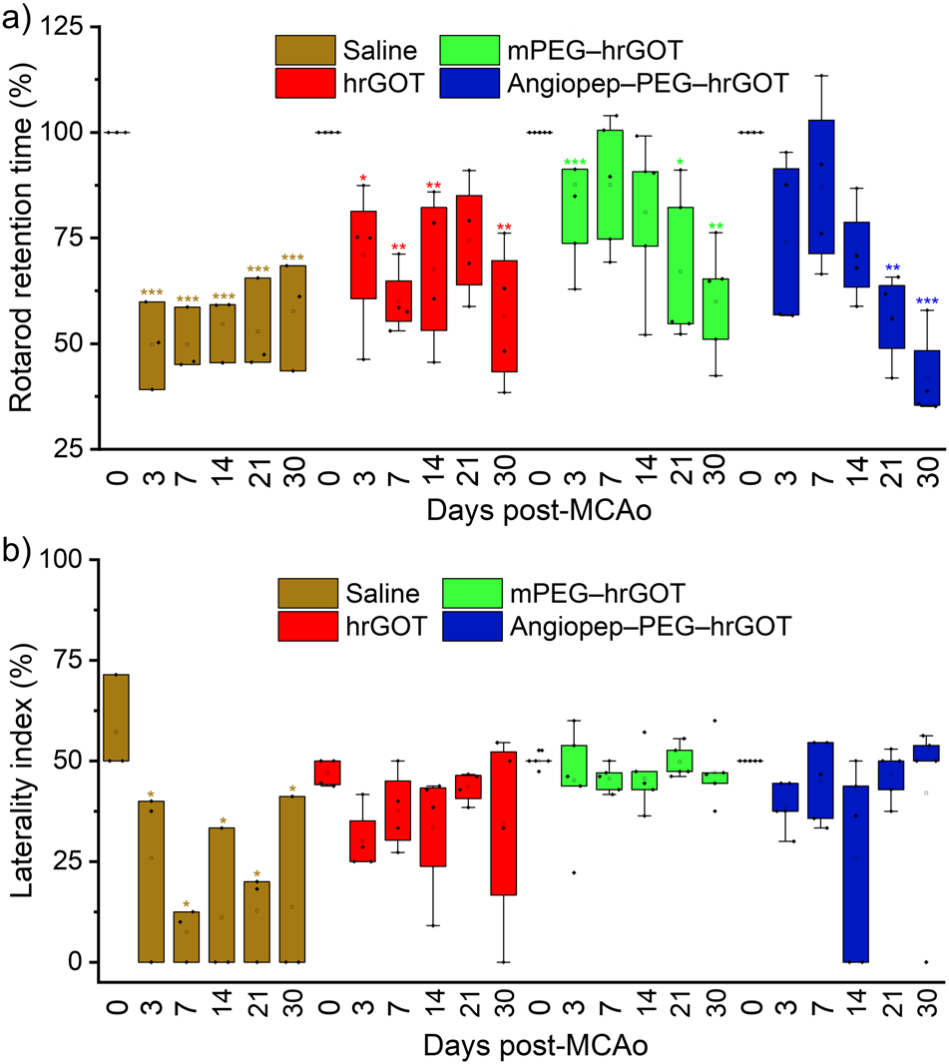
Protective effects assessed by sensorimotor tests. **a** Motor deficits evaluated by the accelerating rotarod test and expressed as the percentage of time that rats remained on the rotarod after ischemia compared with pre-ischemia testing. **b** Sensorimotor deficits assessed using the cylinder test and quantified by laterality index as a function of time after MCAo. Stars denote statistically significant differences with respect to pre-ischemia (repeated measures ANOVA, Bonferroni, *p ≤ 0.05, **p ≤ 0.01, ***p ≤ 0.001). Data presented as mean + SD, *n* = 5.

To confirm the protective properties of the bioconjugates, the motor functions of ischemic rats were assessed using the accelerated rotarod and cylinder tests. The sensitivity of the accelerated rotarod test to detect motor impairments in ischemic models has been well established^21,22^. Prior to surgery, all groups spent the same time on the accelerating rotarod and thus the time spent on the apparatus for the different test groups was expressed as a percentage of pre-surgery values (Fig. 4a). At Day 3, MCAo caused impairment in rotarod performance for the saline and hrGOT-treated groups, though not for the bioconjugate groups. Retention time remained relatively stable for all groups until day 21 or 30, after which it decreased for all groups because the rats likely lost their motivation to perform the physical exercise required to remain on the cylinder (lost their fear of falling), which is one of the main limitations of this test. Intriguingly, performance appeared to degrade to a greater extent for the bioconjugate treated groups compared with the other groups at these late time points. This decline in performance is believed to result from the caveat above rather than from real functional impairment, as it is not seen in the other functional test. Nevertheless, although the data available does not hint towards long term side-effects caused by the sustained depletion of blood glutamate, such a phenomenon cannot be excluded at this stage and should be considered in the context of future toxicological tests. In comparison with the saline group, animals treated with hrGOT bioconjugates displayed a significant increase in the use of contralateral (left) paw (*p* ≤ 0.001). Interestingly, rats having received the bioconjugates also showed improved exploratory activity from Day 3 onwards, as evidenced by an increased amount of rearing. Moreover, there was an increase in left forelimb usage for hrGOT-treated rats compared to control animals at Day 7, 14, and 21 (Fig. 4b). To the best of our knowledge, there is no clear association between functional recovery and the reduction of the lesion size in the literature for the MCAo model employed in this work. For example, several studies have demonstrated that stem cell therapies improve functional outcome without reducing the infarct volume^23–26^. Thus, although the motor tests in the present study mostly reflect the reduction in the volume of the infarct, the possible lack of correlation between these parameters does exist in the literature for this model. Overall, rats that underwent MCAo and were treated with hrGOT bioconjugates had a better recovery than those treated with native hrGOT, which in turn had better recovery than those receiving saline. These combined results confirm that maintaining sustained GOT activity in the blood is an important factor contributing to protection following MCAo.

### Glutamate homeostasis

**Fig. 5:**
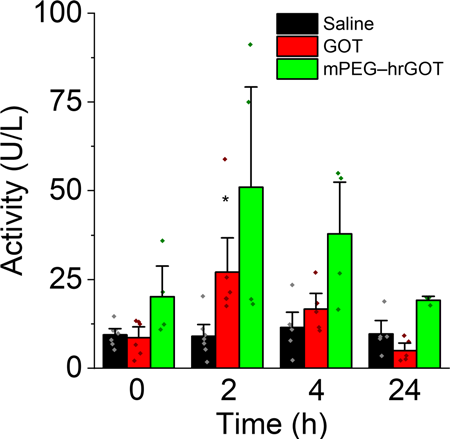
Pharmacokinetics in CSF. Saline, hrGOT, and mPEG–hrGOT were administered to healthy rats. The catalytic activity was monitored in CSF samples taken at specific time points. Stars denote statistically significant differences with respect to basal GOT activity of the CSF. (ANOVA, Tukey, *p < 0.05). Data presented as mean+SD, *n* = 3–5.

At the administered dose of hrGOT, the steady-state concentration of blood glutamate was not significantly affected by any of the treatments, and thus no correlation between enzymatic activity and blood glutamate levels could be identified (Figs. 2a, b and S4a, b). This result might suggest a rapid homeostasis between blood and peripheral tissue. Moreover, similar to the observations in blood, no effect on CSF glutamate concentration was observed (Fig. S5), suggesting once again rapid glutamate homeostasis in the brain. Nevertheless, GOT activity increased very slightly at 2 h in the CSF after administration of the native protein, possibly indicating that a very small portion of the dose is reaching the CSF (Fig. 5). It should be noted, however, that the ~25 U L^–1^ increase observed in the CSF is very small compared with the 3000–6000 U L^–1^ activity observed in the blood. mPEG–hrGOT, in contrast, did not alter GOT activity in the CSF (not statistically significant) suggesting that the presence of the polymer corona prevented passage through the brain or the blood–cerebrospinal fluid barrier. The non-variation of glutamate CSF levels following administration of either hrGOT or mPEG–hrGOT can be rationalized by the glutamate homeostasis system existing in the different parts of the central nervous system including the BBB, the circumventricular organs, and the choroid plexus^27^. Indeed, the BBB as well as the brain–CSF barrier work in concert to maintain and restore the homeostatic balance of neurotransmitters such as glutamate^28^. The pharmacokinetics/pharmacodynamics observed for the targeted (Angiopep–PEG–hrGOT bearing ~22 copies of Angiopep-2) and non-targeted bioconjugates (mPEG–hrGOT) (Figs. 2a, b and S4a, b) were not statistically different from one another, suggesting that brain or near-brain accumulation of the targeted bioconjugate represents only a very minor fraction of the total administered dose and does not contribute significantly to the results.

## Discussion

Preclinical testing criteria for protective agents define a reduction of the ischemic damage by at least 50%, or even better to 80% before it can be considered for clinical trials^29^. In light of the fact that the hrGOT bioconjugates reduced the infarct size by ~70% relative to its Day 0 value, this protective strategy appears very promising. The increase in circulation half-life from hours to days achieved by modifying hrGOT with polymers promoted blood exposure and increased the therapeutic effect. The rapid clearance of hrGOT might be related to its peripheral uptake. Indeed, previous studies have shown that intravenously administrated ^125^I-labeled GOT can be taken up and metabolized by different tissues (liver, spleen, kidney, and intestines). Interestingly, the radioactivity associated with liver and spleen reached its maximum 2 hours post injection and the time course coincided with that for the plasma clearance in the fast phase^30^. The subsequent decrease in radioactivity of these tissues was accompanied by an increase in urinary radioactivity^30^. Therefore, the prolonged blood residence time of the bioconjugates was likely owing to the hindrance of hrGOT uptake by peripheral organs. Moreover, conceivably, the presence of PEG chains around the enzyme surface reduced the immunogenicity of hrGOT (if existent) as well as its rate of proteolytic degradation. Nevertheless, such effects are less prominent than those implicating the liver, spleen, and kidney.

Although measuring lesion size provides some evidence of protection, this parameter differs substantially from functional impairment, which is the most important parameter for patients. MCAo results in a marked deficit in neurological function and motor performance, with animals generally demonstrating weakened limbs. Based on numerous rodent studies reporting that lesion size does not necessarily correlate with functional deficits, lesion size alone is insufficient to characterize the protective character of the hrGOT preparations^22,31–33^. The improvement of the motor functions recorded by the accelerated rotarod test following treatment with the bioconjugates was pronounced compared with other groups, and correlated with those obtained by the cylinder test, though a slight difference in the outcomes of the two sensorimotor tests was noticed. Targeting the hrGOT bioconjugates to the BBB, via the appended Angiopep-2 ligands did not have additional therapeutic value compared to stabilizing and increasing the circulation lifetime of hrGOT using mPEG. This result suggests that glutamate transport across the BBB is not the rate-limiting step to protection. Nevertheless, future studies may investigate strategies to achieve brain accumulation, which is very challenging indeed, or explore methods to overexpress endogenous GOT directly in the brain so as to investigate whether localized GOT treatment offers enhanced protection by outcompeting glutamate homeostasis with peripheral tissue.

Contrary to what has been reported in some studies^11^, a clear effect of hrGOT or hrGOT bioconjugates on serum glutamate levels was not observed. It should be noted that many published works do to mention the specific activity of their hrGOT protein, which make it impossible to compare doses between studies. Future dose escalation studies with the bioconjugates will resolve whether dose is indeed responsible for this discrepancy. Nevertheless, the present results are consistent with the observations from others of a clockwise hysteresis on the pharmacokinetics/pharmacodynamics curves, suggesting that it takes a certain time to deplete peripheral glutamate sufficiently for there to be a noticeable difference in the blood^34^. In general, the homeostasis of glutamate is rapid and maintained through a variety of peripheral transporters and receptors^35,36^, yielding rapid efflux of glutamate towards the blood from organs (e.g., brain) and peripheral tissues. In fact, prior work has shown that the radioactivity of intravenously injected (radioactive) glutamate decayed with a half-life of ~3 min, owing to metabolic reactions occurring in the liver or in the plasma, which provides some insight into the timescale of glutamate homoestasis^37^. This is the most probable explanation for the lack of observable depletion of glutamate in the blood concurrent with neuroprotection in the brain. To investigate whether a more localized effect may be observed, GOT activity and glutamate levels were measured in the CSF of healthy rats receiving either native hrGOT or mPEG–hrGOT. Very little or no effect on the CSF glutamate concentrations was also observed. These results are in accordance with a recently published study showing that the daily administration of recombinant GOT (every 12 hours on days 1–4) reversed the disruption of synaptic plasticity in a rat model of traumatic brain injury by decreasing the glutamate level in hippocampus interstitial fluid, but not ventricular CSF^38^. Overall, these observations provide strong support for the effect of sustained blood glutamate depletion by the bioconjugates on ischemic damage, which merits further investigation. Indeed, these findings raise new questions related to the safety and tolerability of such treatments. Indeed, new protection or healing mechanisms (yet to be identified) may be involved in addition to those expected for hrGOT. Overall, glutamate scavenging strategies are highly suited to overcome many of the drawbacks of the receptor-based therapies (e.g., cognitive impairments, hallucinations, and even coma)^39^ and this concept is an attractive protective strategy to remove excess glutamate in the brain interstitium. The most significant observation of this study is that maintaining high and relatively stable hrGOT blood activity over a period of several days had a very beneficial effect on infarct volume as well as sensorimotor function^11,40–42^. This was achieved with a single administration of hrGOT bioconjugate. Remarkably, equivalent doses of native hrGOT alone yielded results equivalent to saline controls for certain tests, and did not alter blood or CSF glutamate levels. Overall, the efficacy of this strategy holds great potential as a therapy for stroke^43^, as well as other pathologies associated with acute excitotoxicity such as spinal cord injury^44^ or traumatic brain injury^38^.

## Materials and methods

### Reagents

Amicon ultra centrifugal filter units (molecular weight cutoff [MWCO] 30 kDa), dimethyl sulfoxide, potassium phosphate monobasic, potassium phosphate dibasic, sodium tetraborate decahydrate, sodium acetate, o-pthaldialdehyde, 2-mercaptoethanol, and deuterium oxide were purchased from Sigma Aldrich (Oakville, Canada). α-Methoxy, ω-succinimidyl carboxymethyl ester poly(ethylene glycol) (mPEG–NHS; 5 kDa) and α-maleimide, ω-succinimidyl carboxymethyl ester PEG (Mal–PEG–NHS; 5 kDa) were purchased from JenKem Technology (Plano, USA). Aspartate Aminotransferase Activity Assay Kits were purchased from Abcam (Cambridge, UK). Angiopep (TFFYGGSRGKRNNFKTEEYC; 2.4 kDa) was purchased from Zhejiang Ontores Biotechnologies (China). Mini-PROTEIN TGX Stain-Free Precast Gels (4–15% acrylamide) and Bio-Rad precision plus protein unstained standards were purchased from Bio-Rad (Saint-Laurent, Canada). BD Microtainer^®^ blood collection SST tubes and BD IV catheter Insyte 24GA × 0.75” YLW BX/50 were purchased from Becton Dickinson (New Jersey, USA). All buffers were prepared using MilliQ water (mean resistivity 18.2 MΩ cm). All chemicals were purchased at the highest grade possible and used as received. hrGOT (∼1 kU/mg) was used.

### Synthesis of mPEG–hrGOT and angiopep–PEG–hrGOT

To prepare mPEG–hrGOT, a 3-mL solution of hrGOT (7 mg mL^–1^) was prepared in potassium phosphate buffer (100 mM, pH 7.2). To this solution was added mPEG–NHS (50 eq.) and the reaction mixture was mildly stirred for 30 min at room temperature in a sealed glass vial. After this period, mPEG–hrGOT was purified by size-exclusion chromatography (SEC) using an ÄKTA Start fast protein liquid chromatographer (FPLC) equipped with a HiPrep 16/60 Sephacryl™ S200 HR column (Fig. S1a). Filtered (0.2 µm) potassium phosphate buffer (100 mM, pH 7.2) was used to elute samples at a flow rate of 0.8 mL min^–1^. The column was equilibrated for 0.2 column volumes (CV) before sample injection and elution occurred over 120 mL (1 CV) while continuously collecting 4 mL fractions. To prepare Angiopep–PEG–hrGOT, hrGOT was modified with Mal–PEG–NHS and purified according to the procedure above. Following purification by SEC, the collected fractions containing Mal–PEG–hrGOT (Fig. S1b) were concentrated by centrifugal dialysis (MWCO 30 kDa) and added directly to a vial containing Angiopep (2 eq. relative to the expected amount of Mal on the conjugate). The solution was left for 30 min at room temperature, then incubated for 24 h at 4 °C in the dark. Angiopep–PEG–hrGOT was isolated from residual Angiopep by centrifugal dialysis (MWCO 30 kDa) at 5000 × *g* for 60 min at 4 °C. The conjugates were stored frozen at –20 °C until used.

To determine the degree of PEGylation of the bioconjugates, SDS–PAGE was performed. hrGOT (5 µg in 3 µL water) and hrGOT bioconjugates (5 µg protein in 3 µL water) were mixed with 5 µL of loading buffer (65 mM Tris-HC1, pH 6.8, 2% SDS, 10% glycerol, and 0.1% bromophenol blue). Gels were run in Tris/glycine/SDS (3.0/14.4/1.0 g L^–1^) buffer pH 8.3, under constant voltage (100 V) for ~90 min. The gels were imaged using an UVP Biodoc-it imaging system running UVP VisionWorksLS™ software.

The degree of PEGylation was determined by ^1^H NMR spectroscopy using a Bruker Av300 spectrometer operating at 300 MHz for protons, as described elsewhere^45^. This parameter was also determined by UV-Vis spectroscopy. For this, a known mass of bioconjugate was dissolved in a known volume of distilled water. The number of moles of the hrGOT component of the bioconjugate in the solution was determined via the absorbance at 280 nm and the extinction coefficient of hrGOT (140,000 M^−1^ cm^−1^). Dividing the mass of the bioconjugate by the number of moles of the hrGOT component yields the molecular weight of the bioconjugate, from which can be determined the degree of PEGylation ([MW_Bioconjugate_ (kDa) – MW_hrGOT_ (kDa)] ÷ MW_PEG_ (5 kDa)). mPEG does not absorb significantly at this wavelength and thus does not interfere with quantification of molecular weight in this manner.

The molecular weight distribution of the bioconjugates was analyzed by aqueous SEC using an Agilent Technologies 1260 HPLC equipped with two Agilent Bio-SEC5 columns mounted in series (5 µm, 7.8 × 300 mm), with 1000 Å and 2000 Å nominal pore sizes, a 1200 Infinity photodiode array detector VL, and a 1290 Infinity II evaporative light scattering detector. One hundred-µL of a 0.5 mg mL^–1^ sample solution prepared in ammonium formate buffer (100 mM, pH 3.5) was injected and eluted with ammonium formate buffer (100 mM, pH 3.5) at 1 mL min^–1^ at 25 °C.

### Animals, surgical procedures, and inclusion criteria

Experimental protocols were approved by the local Animal Care Committee according to the requirements of the European Union (86/609/CEE, 2003/65/CE and 2010/63/EU). Male Sprague–Dawley rats (Harlan Laboratories, Barcelona, Spain) weighing 250–300 g (8–10 weeks old) were housed individually at an environmental temperature of 23 °C with 40% relative humidity and had a 12 h light–dark cycle. They were watered and fed ad libitum. All surgical procedures were performed under sevoflurane anesthesia (6% induction and 4% maintenance in a mixture of 70% NO_2_ and 30% O_2_). Rectal temperature was maintained at 37 ± 0.5 °C during surgery using a thermostat-controlled electric pad (Neos Biotec, Pamplona, Spain).

Transient focal ischemia was induced in rats by transient middle cerebral artery occlusion (MCAo) following surgical procedures previously described^11,46^. In brief, under an operating microscope, following a midline neck incision, the left common, the external, and the internal carotid arteries were separated from the connective tissues. Using 5–0 silk sutures, the left external carotid artery as well as the pterygopalatine artery of the internal carotid were ligated. A silicon rubber-coated size 4–0 monofilament (diameter 0.19 mm, length 23 mm; diameter with coating 0.37 ± 0.02 mm; coating length 3–4 mm) (Doccol Corporation, Sharon, MA) was inserted into the stump of the left common carotid artery and advanced into the internal carotid artery to 20 mm from the bifurcation to occlude the origin of the MCA. The suture was removed after 75 min of occlusion. A laser Doppler flow probe (tip diameter 1 mm) attached to a PeriFlux 5000 Laser Doppler Flowmeter (Perimed AB, Stockholm, Sweden) was placed over the thinned skull in the MCA territory (4 mm lateral to bregma) to obtain a continuous measure of relative cerebral flow during the experiment. Occlusion and reperfusion were monitored by laser doppler monitoring and by diffusion weighed imaging (DWI) by magnetic resonance imaging (MRI). In combination with DWI, magnetic resonance angiography (MRA) was performed to ensure that the artery remained occluded throughout the procedure and to confirm the exclusive occlusion of the MCA. Ischemic lesions were determined from T_2_ maps, according to the MCAo model described in our previous study^47^.

Experimental procedures were performed following five criteria derived from the Stroke Therapy Academic Industry Roundtable^48^ group guidelines for preclinical evaluation of stroke therapeutics: (1) cerebral serum flow was monitored to confirm the vascular occlusion as an index of the reliability of the ischemic model; (2) animals were randomly assigned to treatment groups of the study; (3) researchers were blinded to treatment administration; (4) researchers were blinded to treatments during result assessment; and (5) temperature was controlled during the ischemic period. The only animals included in this study had: (1) cerebral serum flow reduction of >70% by laser Doppler monitoring; (2) DWI hemispheric infarct volume between 30% and 45% (indicated as the percentage of ischemic damage with respect to the ipsilateral hemisphere volume); (3) MRA of the MCAo; and (4) complete reperfusion (>60%) after MCAo.

In this study, a total of 46 animals were included. Four animals were excluded because of bleeding and spontaneous death during surgery. Based on doppler monitoring, the remaining animals (*n* = 42) had successful MCAo (>70% of the cerebral blood flow with respect to the basal level). However, when these animals were analyzed by MRA during arterial occlusion, 13 were excluded because both the MCA and the anterior cerebral artery had been occluded. Moreover, when DWI was performed on the remaining 29 animals, 9 of them were excluded because the infarcted regions were out of the established range (35–45%). The DWI volume of 20 rats that were ultimately included in the study was 41.3 ± 5.4%.

### Pharmacokinetics and pharmacodynamics in healthy rats

Groups of three healthy rats were administered 1 mg kg^−1^ (protein equivalent) of hrGOT, mPEG–hrGOT, or angiopep–PEG–hrGOT in 1 mL sterile saline by intravenous injection via the tail vein. Control groups (*n* = 3 each) received either 1 mL of saline (0.9% of NaCl) or 1.3 mg kg^−1^ mPEG (equivalent to the amount administered for the bioconjugates, vide infra). Approximately 300 µL of blood was sampled from the tail vein repeatedly over a period of one month (1, 2, 4, and 6 h, 1, 2, 4, 6, 8, 14, 21, and 30 days). To investigate the efflux hypothesis, CSF was collected from rats treated with 1 mg kg^−1^ (protein equivalent) hrGOT, mPEG–hrGOT, or sterile saline (control). The CSF was obtained from the cistern magna and was carried out using the protocol described by Pegg et al.^49^. In brief, using the occipital crest as a reference point, a midline incision was made beginning between the ears and ending ~2 cm caudally. The fascia was retracted and muscles were dissected until the cistern magna was exposed, which appeared as a tiny inverted triangle, outlined by the cerebellum above and the medulla below, behind the translucent dural membrane. Once the cistern magna was identified, a glass capillary was inserted and a volume of 3–5 µL of CSF was collected at every puncture. CSF was collected at different time points, transferred to a tube, and kept frozen at –80 °C until used.

### Pharmacokinetics in ischemic rats and protective study

Rats were randomly attributed to one of four experimental groups (*n* = 5 each): Saline, hrGOT (1 mg kg^−1^ protein), mPEG–hrGOT (1 mg kg^−1^ protein), or Angiopep–PEG–hrGOT (1 mg kg^−1^ protein). Treatments were administered via tail vein injection immediately after reperfusion. The serum GOT activity was determined under basal conditions (before surgery) and at different times after reperfusion (1 and 6 h, 1, 2, 3, 4, 6, 8, 14, 21, and 30 days). T_2_-weighted (T_2_W) images were acquired at different time points (1, 3, 7, 14, 21, and 30 days) after the onset of ischemia. Recovery of functionality was studied by means of the cylinder and accelerated rotarod tests, which were carried out under basal conditions (before surgery) and 3, 7, 14, 21, and 30 days after ischemia.

### Analysis of GOT activity in serum and CSF

Blood samples were collected in test tubes and immediately centrifuged at 3000 rpm for 7 min for collection of serum that was stored frozen (−80 °C) until analyzed. GOT activity, in serum and CSF, was determined by means of an Aspartate Aminotransferase activity assay kit (Abcam, Cambridge, UK) following the manufacturer’s recommended protocol.

### Monitoring of glutamate concentration in serum and CSF by HPLC

The concentration of glutamate was determined using a pre-column derivatization high performance liquid chromatography (HPLC) method. For serum, samples (7.5 μL) were deproteinized with 30 μL ice-cold methanol. The solution was vortexed and then centrifuged at 20,000 × *g* for 5 min at 4 °C. The pellet was discarded and 25 μL of the supernatant was collected and mixed with 5 μL 20% SDS in water and 25 μL 0.1 M sodium tetraborate (pH 9.5). Thereafter, 50 μL o-pthaldialdehyde/2-mercaptoethanol derivatization solution (freshly prepared by dissolving 50 mg of o-pthaldialdehyde in 1.25 mL of absolute methanol, followed by the addition of 50 μL of 2-mercaptoethanol and 11.2 mL of 0.1 M sodium tetraborate, pH 9.5) was added and mixed thoroughly. Subsequently, 50 μL of 1 M sodium acetate (pH 7.2) was added and the mixture was injected onto the equilibrated HPLC column. For the CSF, the same volume ratios were followed using a sample volume of 3.5 μL. A gradient elution of 40 mM NaH_2_PO_4_ (pH 7.8) and acetonitrile: methanol: water (45:45:10, v/v/v) with a flow rate of 2 mL min^–1^ was employed. The gradient was (% 40 mM NaH_2_PO_4_) (% acetonitrile: methanol: water): 0 min (100), 1.9 min (100), 18.1 min (43), 18.6 min (0), 22.3 min (0), 23.2 min (100), and 26 min (100). Analytes were separated at 30 ± 2 °C on a ZORBAX Eclipse AAA C_18_ reverse-phase column (4.6 × 150 mm, 3.5 µm) and detected by fluorescence (*λ*_ex_ = 340 nm and *λ*_em_ = 450 nm). A glutamate standard curve was established with concentrations ranging between 0 and 400 μM (Fig. S2). All determinations were performed at least in duplicate. A typical HPLC chromatogram is shown in Fig. S3. Note: minimal loss of glutamate was observed during protein precipitation by comparing precipitation using different organic solvents and acids as well as ultrafiltration (instead of protein precipitation); and bioconjugates were also removed during this step, owing to their absence in the chromatograms used for glutamate quantification.

### Magnetic resonance imaging and image analysis

MRI studies were conducted on a 9.4 T horizontal bore magnet (Bruker BioSpin, Ettligen, Germany) with 12-cm wide actively shielded gradient coils (440 mT m^–1^). Radiofrequency transmission was achieved with a birdcage volume resonator and signal was detected using a four-element arrayed surface coil positioned over the head of the animal. The latter was fixed with a teeth bar, earplugs, and adhesive tape. Transmission and reception coils were actively decoupled from each other. Gradient–echo pilot scans were performed at the beginning of each imaging session for accurate positioning of the animal inside the magnet bore. Apparent diffusion coefficient (ADC) maps were acquired during MCAo (75 min after the onset of ischemia) from DWI using a spin echo echo-planar imaging sequence with the following acquisition parameters: echo time = 24.89 ms, repetition time (RT) = 4.5 s, spectral bandwidth (SW) = 200 kHz, seven *b* values of 0, 300, 600, 900, 1200, 1600, and 2000 s mm^–2^, flip angle (FA) = 90°, number of averages (NA) = 3, 14 consecutive slices of 1 mm, 24 × 16 mm^2^ field of view (FOV) (with saturation bands to suppress signal outside this FOV), a matrix size of 96 × 64 (isotropic in-plane resolution of 250 μm pixel^–1^ × 250 μm pixel^–1^) and implemented with fat suppression option. MCAo status was evaluated in a non-invasive manner with the time-of-flight MRA (TOF-MRA). The TOF-MRA scan was performed with a 3D-Flash sequence with an ET = 2.8 ms, RT = 15 ms, FA = 30°, NA = 2, SW = 98 kHz, 1 slice of 14 mm, 30.72 × 30.72 × 14 mm^3^ FOV (with saturation bands to suppress signal outside this FOV), a matrix size of 256 × 256 × 58 (resolution of 120 μm pixel^–1^ × 120 μm pixel^–1^ × 241 μm pixel^–1^) and implemented without fat suppression option. The progression of ischemic lesions and infarct volumes were determined from T_2_-maps calculated from T_2_W images acquired 1, 7, 14, 21, and 30 days after the onset of ischemia using a MSME sequence with an ET = 9 ms, RT = 3 s, 16 echoes with 9 ms echo spacing, FA = 180°, NA = 2, SW = 75 kHz, 14 slices of 1 mm, 19.2 × 19.2 mm^2^ FOV (with saturation bands to suppress signal outside this FOV), a matrix size of 192 × 192 (isotropic in-plane resolution of 100 μm pixel^–1^ × 100 μm pixel^–1^) and implemented without fat suppression option. All images were processed, and maps were constructed with ImageJ (https://imagej.nih.gov/ij/). Infarct volumes were determined from ADC maps and T_2_ relaxation maps by manually selecting areas of reduced ADC values or hyperintense T_2_ signal by a researcher blinded to the animal protocols^16^. Infarct size was indicated as the percentage of ischemic damage with respect to the ipsilateral hemispheric volume, corrected for brain edema. For each brain slice, the total areas of both hemispheres and the areas of infarction were calculated. An edema index was measured by quantifying the midline deviation (MD) calculated as the ratio between the volume of the ipsilateral hemisphere and the volume of the contralateral hemisphere. The actual infarct size was adjusted for edema by dividing the area of infarction by the edema index [mm^3^/MD]. Thereafter, the presented infarct volume was calculated as following: (infarct volume [mm^3^/MD]/ipsilateral hemispheric area [mm^3^]) × 100. These procedures have been used repeatedly in the literature to measure and evaluate stroke outcome in experimental models.

### Motor and somatosensory tests

To examine the effect of hrGOT, mPEG–hrGOT, and angiopep–PEG–hrGOT on functional outcomes of ischemic rats, a battery of behavioral tests was performed pre-MCAo and at 3, 7, 14, 21, and 30 days post-MCAo by an independent investigator blinded to the experimental groups. All tests were performed during the darkness cycle of animal housing^21,22^.

For the cylinder test, somatosensory deficits were evaluated by examination of the asymmetry of limbs during exploratory activity. For this test, rats were introduced into a Plexiglas cylinder (diameter 20 cm; height 40 cm) and a video camera located under this transparent cylinder was used to record the vertical exploratory movement of the rats during 5 min. For the analysis, the VirtualDub software was used and, during slow-motion video playback, instances of the sole use of the ipsilateral or contralateral forelimb or the simultaneous bilateral use of both forelimbs for upright support were recorded. Following each forelimb placement, the subsequent movements (such as lateral exploration) were not scored until the rat returned to the ground, the next placement was then scored. Forelimb contacts while rearing up were scored with a total of 10 contacts recorded for each animal. Laterality index was calculated as following: (number of times that the animal touches the cylinder with the impaired forelimb during the ascendant movement divided by the number of total forelimb contacts) ×100. This index is close to 50% for healthy animals and tends to be 0 or 100% for animals that have a preferential use of the left or the right paw, respectively.

The accelerating rotarod test was performed using rotarod apparatus (47650, Ugo Basile, Comerio, Italy) to evaluate motor balance and coordination impairment. Before surgery, the animals were pre-trained for 7 consecutive days (each animal received three training sessions per day). Rats were placed on the rotarod and the speed of the spindle was slowly increased from 5 to 40 rpm over a period of 5 min. All animals were required to stay on the accelerating rotarod for a minimum of 180 seconds. If they were unable to reach this criterion, the trial was repeated for a maximum of five times instead of three times. Animals achieving the baseline criteria were included for the subsequent study. After surgery, the time that an animal was able to hold itself on the spindle was recorded as the latency to fall. The average of the three fall latency values was used for analysis and the motor test data were presented as percentage of mean latency compared with the internal baseline (prior to surgery, considered as 100%).

### Statistics and reproducibility

Results from pharmacokinetic data were compared by one-way analysis of variance (ANOVA) followed by a Tukey post hoc test. Infarct volume and motor tests data were compared by repeated measures ANOVA followed by a Bonferroni post hoc test. *p* values considered for statistically significance are identified in relevant figure legends. Full pairwise means comparison tables obtained from these tests can be found in the Supporting Information. The raw data that support the findings of this study are available in Table S4–S9 of the Supplementary Information.

### Reporting summary

Further information on research design is available in the Nature Research Reporting Summary linked to this article.

## Supplementary information

Supplementary Results and Data

Reporting Summary
