# Supplementary material for: Sustained blood glutamate scavenging enhances protection in ischemic stroke
Source: Commun Biol. 2020 Dec 3;3:729. doi: 10.1038/s42003-020-01406-1 (PMC7713697; doi:10.1038/s42003-020-01406-1)
Supplement: Supplementary file 1 — Supplementary Results and Data [file 42003_2020_1406_MOESM1_ESM.pdf]

Electronic Supporting Information

for

Sustained blood glutamate scavenging enhances protection in ischemic stroke

Ahlem Zaghmi,[1] Antonio Dopico-López,[2] María Pérez-Mato,[2,3] Ramón Iglesias-Rey,[2] Pablo Hervella,[2] Andrea A Greschner,[1] Ana Bugallo-Casal,[2] Andrés da Silva,[2] María Gutiérrez-Fernández[3,] José Castillo,[2,] Francisco Campos Pérez,[2,*] and Marc A. Gauthier[1,*]

[1] Institut National de la Recherche Scientifique (INRS), EMT Research Center, Varennes, Qc, J3X 1S2, Canada

[2] Clinical Neuroscience Research Laboratory, Health Research Institute of Santiago de Compostela (IDIS), Santiago de Compostela, Spain.

[3] Neuroscience and Cerebrovascular Research Laboratory, Department of Neurology and Stroke Center, La Paz University Hospital, Neuroscience Area of IdiPAZ Health Research Institute, Universidad Autónoma de Madrid, Madrid, Spain.

Corresponding authors:

Marc A. Gauthier
Institut National de la Recherche Scientifique (INRS), EMT Research Center, 1650 boul. Lionel-Boulet, Varennes, J3X 1S2, Canada
E-mail: gauthier@emt.inrs.ca
Telephone: +1 514 228 69 32

Francisco Campos Pérez
Clinical Neuroscience Research Laboratory, Hospital Clínico Universitario, Travesa da Choupana s/n, 15706 Santiago de Compostela, Spain
Email: francisco.campos.perez@sergas.es
Phone: +34 981951097
Fax: +34 981951086

Conflict of interest: None to declare

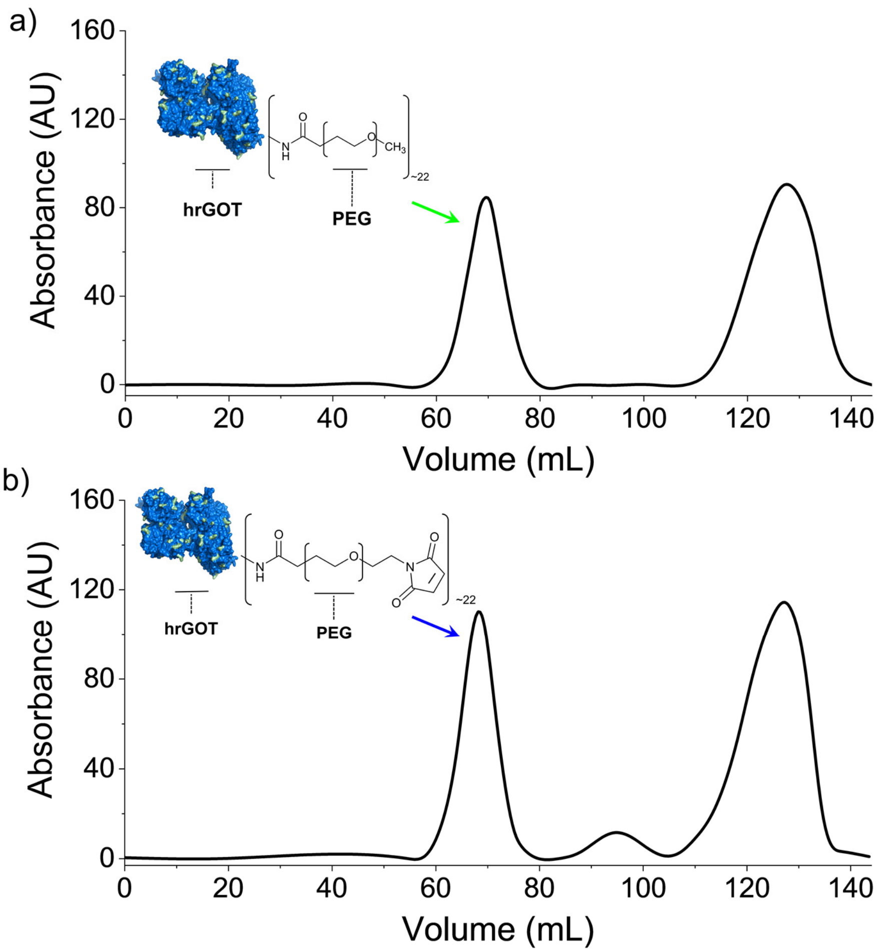

Figure S1| **FPLC chromatograms for the purification of (a) mPEG–hrGOT and (b) Mal–PEG–hrGOT**. Size exclusion chromatography was used to purify the bioconjugates after each reaction (peaks corresponding to mPEG–hrGOT and Mal–PEG–hrGOT are identified).

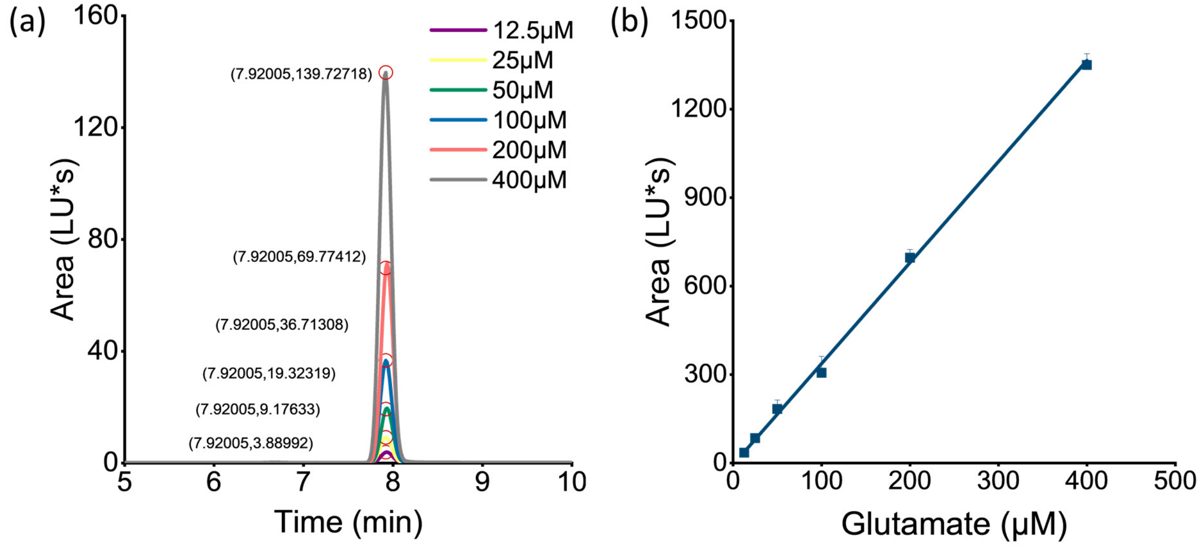

Figure S2| Quantification of Glutamate in serum by HPLC. (a) Representative chromatograms of glutamate (pre-column derivatized with o-pathaldialdehyde/2-mercaptoethanol) at different concentrations. (b) Calibration curves for glutamate.

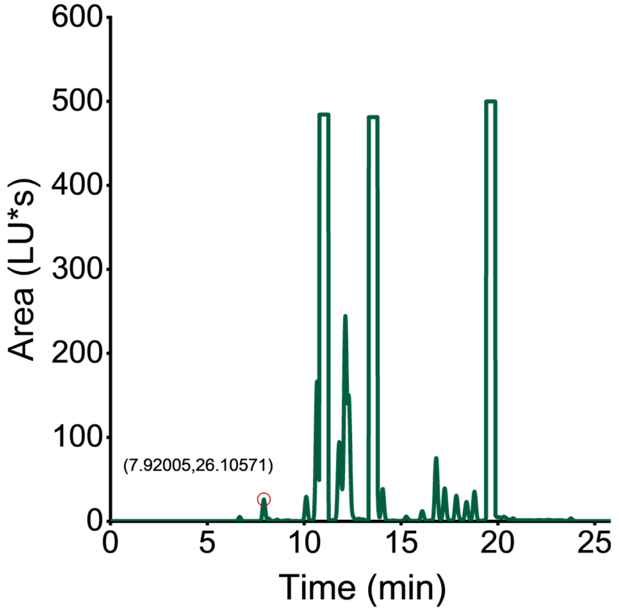

Figure S3| Representative chromatogram of serum (pre-column derivatized with o-pathaldialdehyde/2-mercaptoethanol). Fluorescence detection (λ_{ex} = 340 nm ; λ_{em} = 450 nm). Peak of glutamate identified.

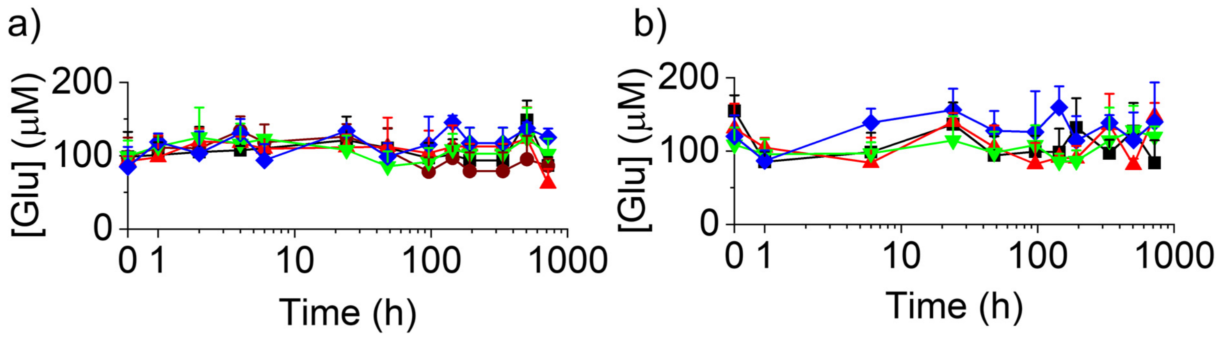

Figure S4| Effects of native hrGOT, mPEG–hrGOT, and Angiopep–PEG–hrGOT on serum glutamate levels in (a) healthy and (b) MCAo rats. No change in steady-state blood glutamate concentrations was observed following intravenous administration in rats. Data presented as Mean + SD, n = 3–5.

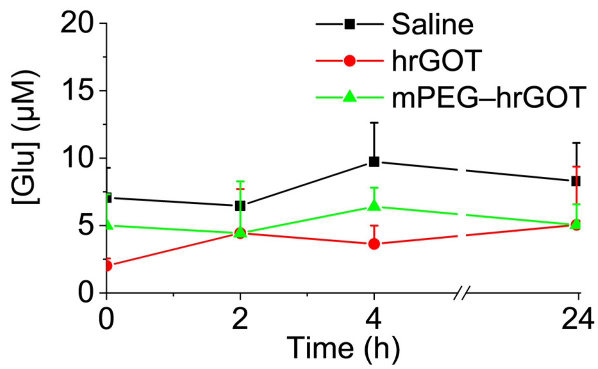

Figure S5| Effects of native hrGOT, mPEG–hrGOT, and Angiopep–PEG–hrGOT on CSF glutamate levels in healthy rats. The glutamate levels were monitored in CSF samples taken at specific time points. Data presented as Mean + SD, n = 3–5.

Table S1. Solvent-accessibility of the lysine residues in hrGOT. Calculated using the PDBePISA (Proteins, Interfaces, Structures and Assemblies) tool on http://www.ebi.ac.uk/msd-srv/prot_int, using PDB 3ii0.

**Lysine residue**	**Degree of accessibility of residue on monomers 1–2 (Å^2)**	
	1	2
6	114.19	114.31
19	57.83	57.42
41	119.16	116.69
42	93.84	94.65
46	115.58	112.54
83	144.05	143.32
85	85.31	152.84
116	60.19	62.76
140	155.38	153.29
152	104.20	79.79
193	76.49	98.74
200	64.43	76.20
245	31.24	29.45
262	124.65	126.17
276	81.82	79.99
311	90.74	89.94
332	164.97	164.25
355	46.27	69.25
364	28.43	27.38
382	126.24	127.80
397	142.26	141.95
405	126.84	123.39

Table S2. Pharmacokinetic parameters (half-life (t½) and Area under the curve (AUC)) of native hrGOT, mPEG–hrGOT, and Angiopep–PEG–hrGOT following intravenous administration in rats (data extracted from Figure 2).

	**Circulation t½ (h)**	**Elimination t½ (h)**	**AUC ((U/L) × h))**
hrGOT	2	6	52542
mPEG–hrGOT	92	192	843805
Angiopep–PEG–hrGOT	92	192	866182

Table S3. Representative ADC maps (Day 0) and T2-weighted images (Day 1 –30) of the 14 consecutive coronal rat brain slices (hind brain to fore brain) from each group at each time point investigated. Regions of interest including the motor cortex (M1/M2), the primary sensory cortex (S1), and the secondary sensory cortex (S2) are identified are identified on one image for illustrative purposes. Note the background was subtracted from Slices 7 and 8 for use as display items in Figure 3 of the Main article.

Slice 1	Slice 2	Slice 3	Slice 4	Slice 5	Slice 6	Slice 7
Slice 8	Slice 9	Slice 10	Slice 11	Slice 12	Slice 13	Slice 14

**Saline**	
Day 0	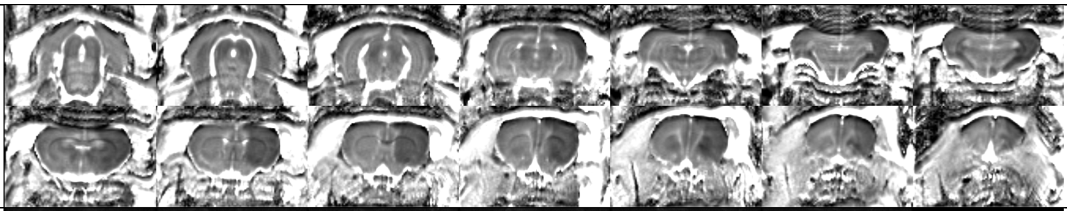
Day 1	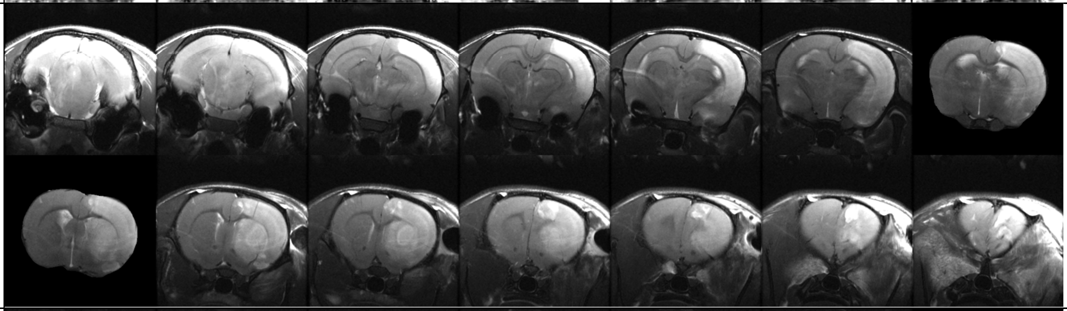
Day 7	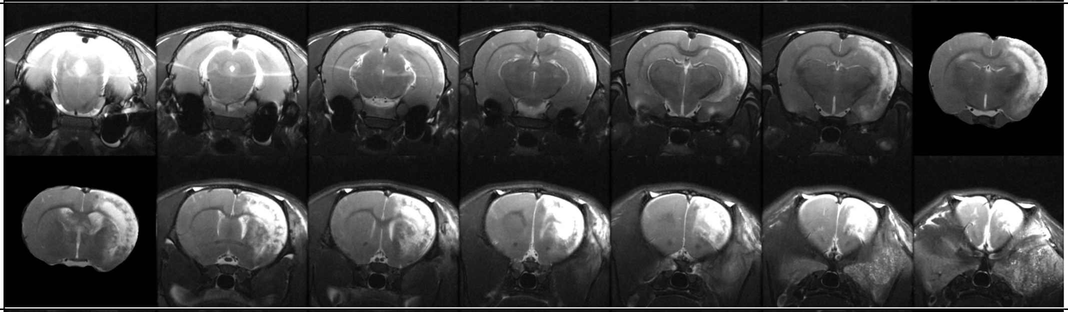
Day 14	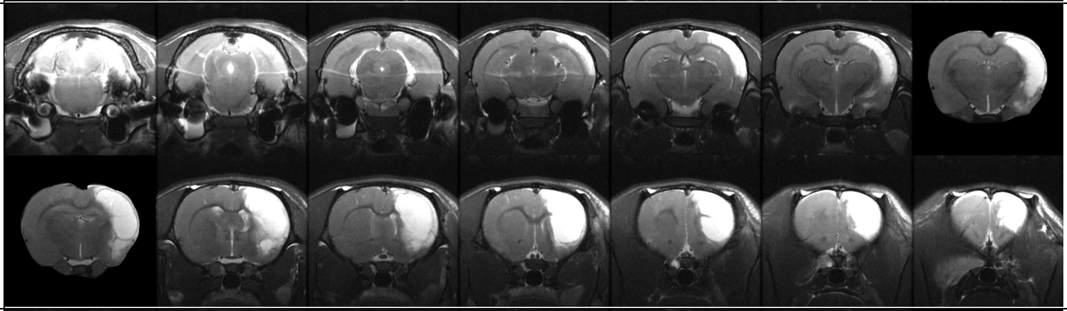
Day 21	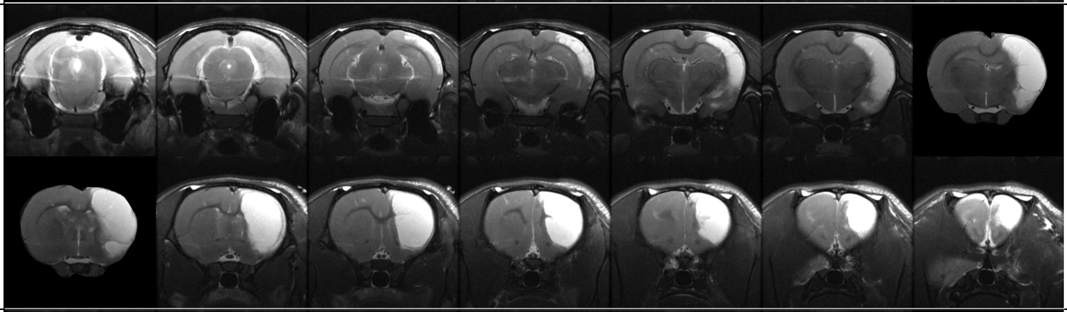
Day 30	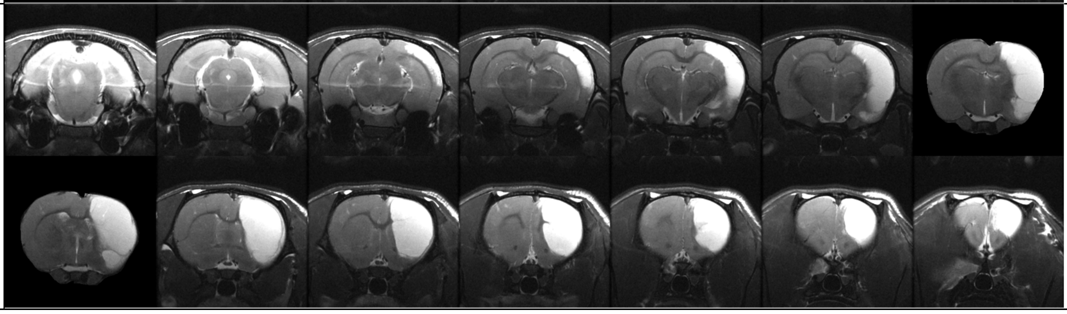

**hrGOT**	
Day 0	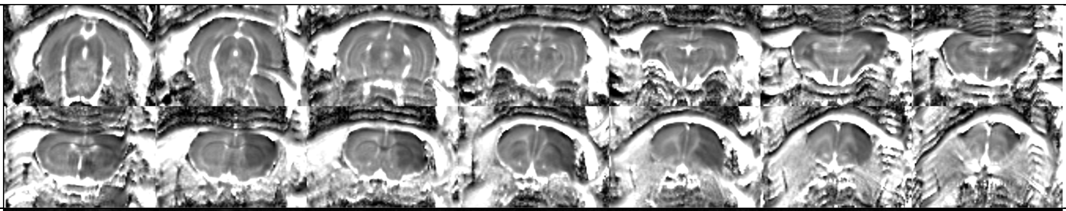
Day 1	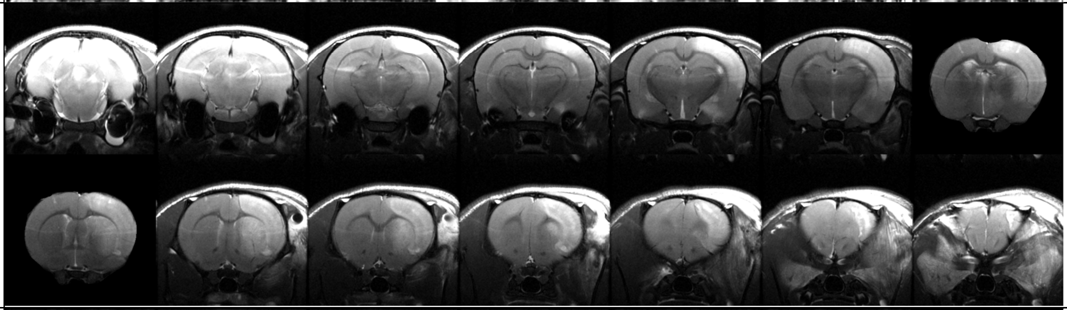
Day 7	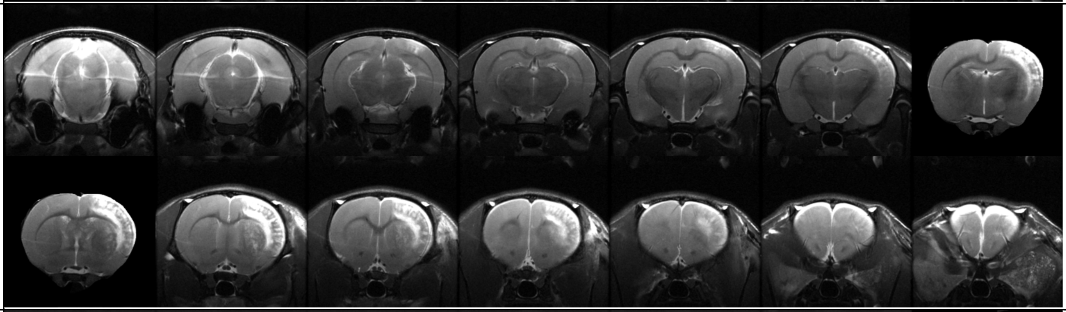
Day 14	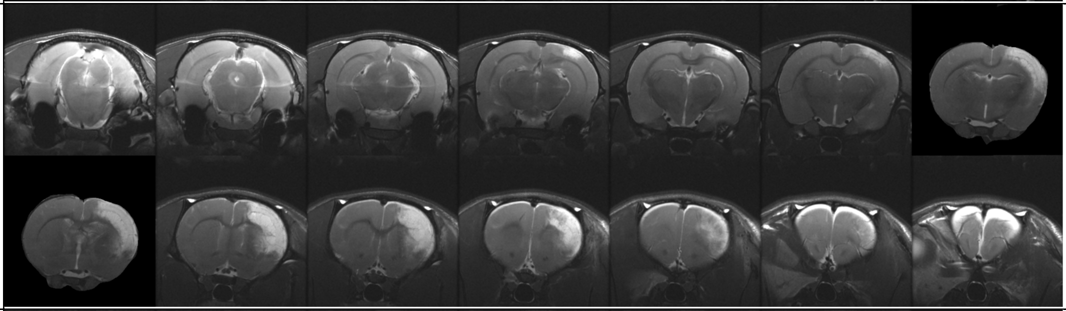
Day 21	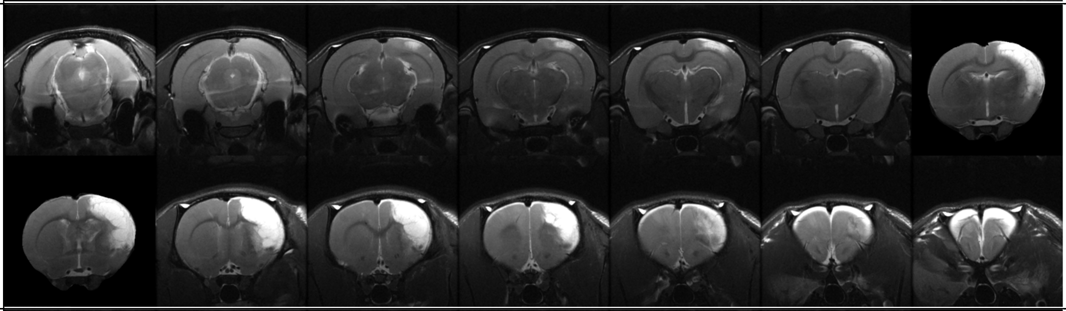
Day 30	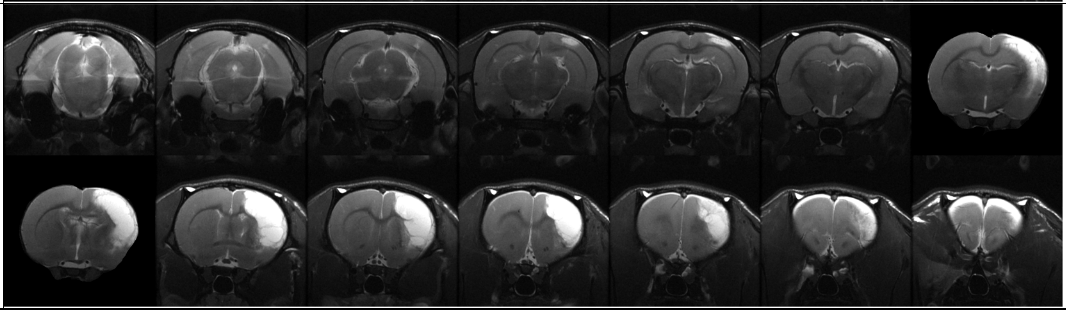

**mPEG–hrGOT**	
Day 0	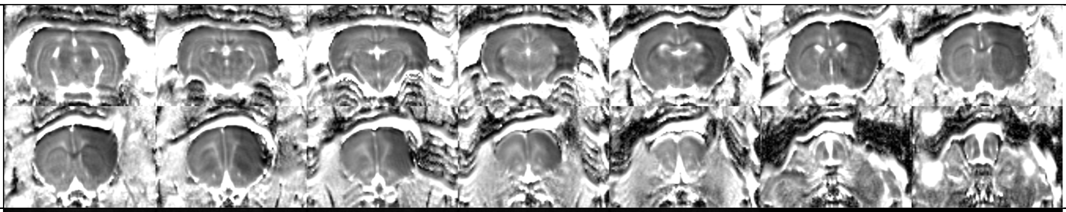
Day 1	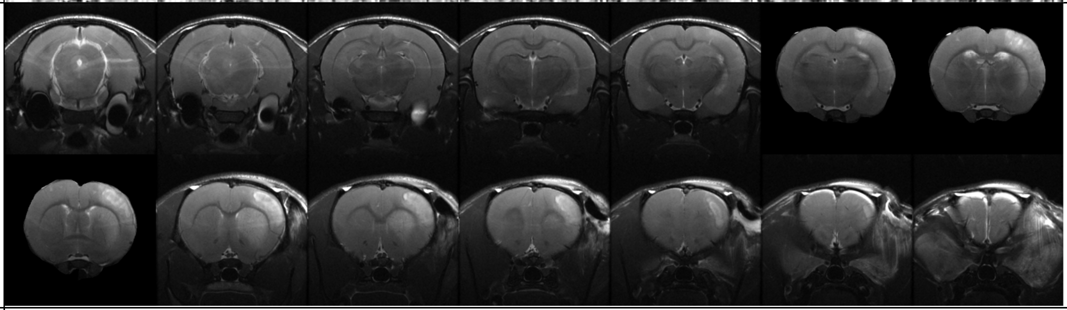
Day 7	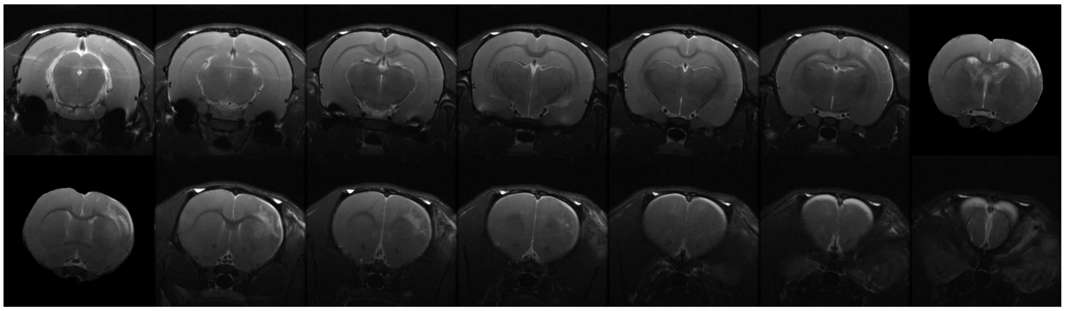
Day 14	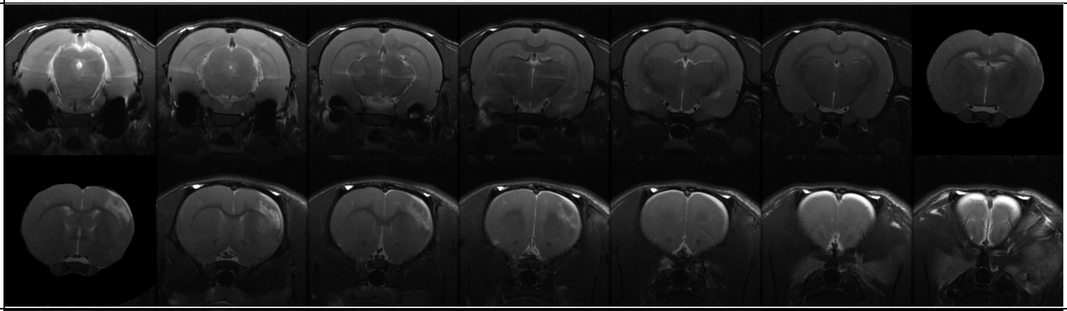
Day 21	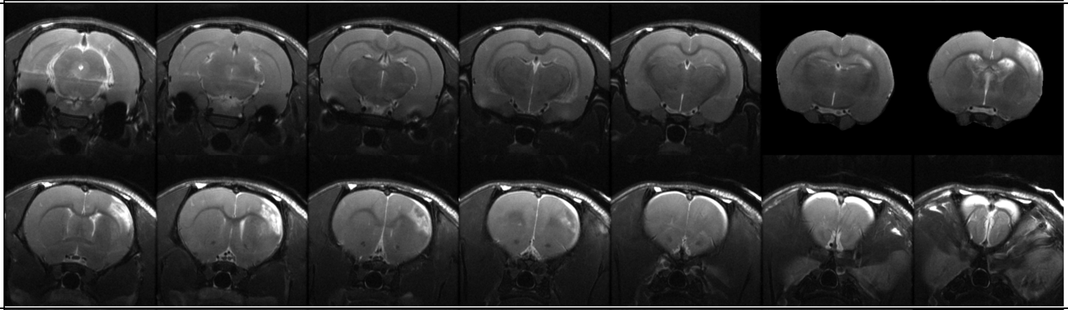
Day 30	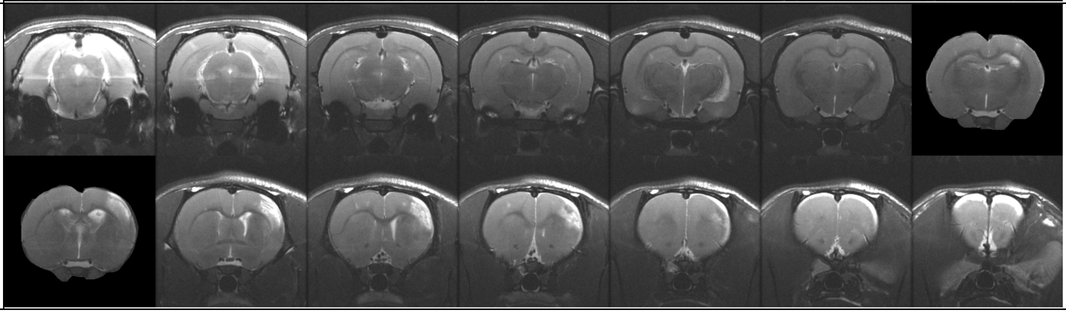

**mPEG–hrGOT**	
Day 0	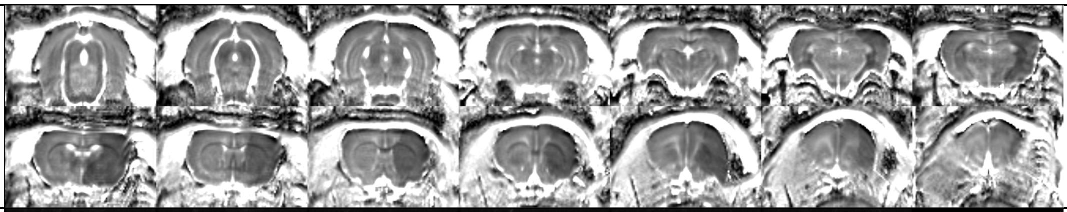
Day 1	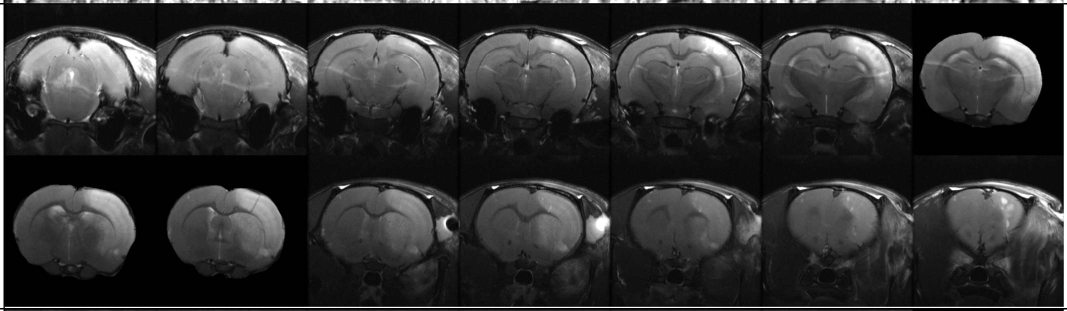
Day 7	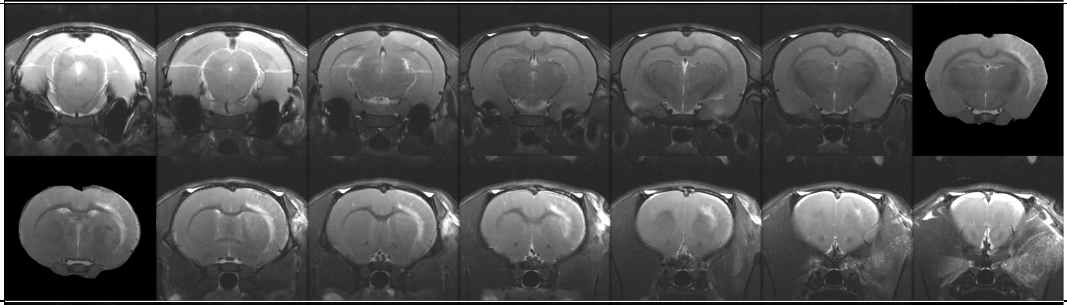
Day 14	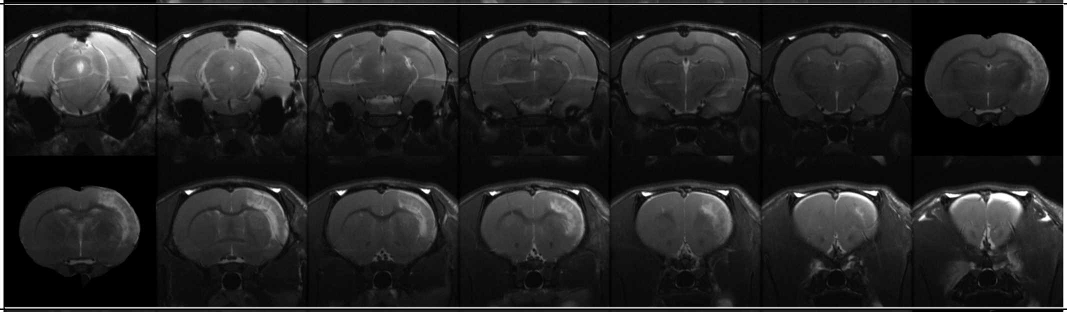
Day 21	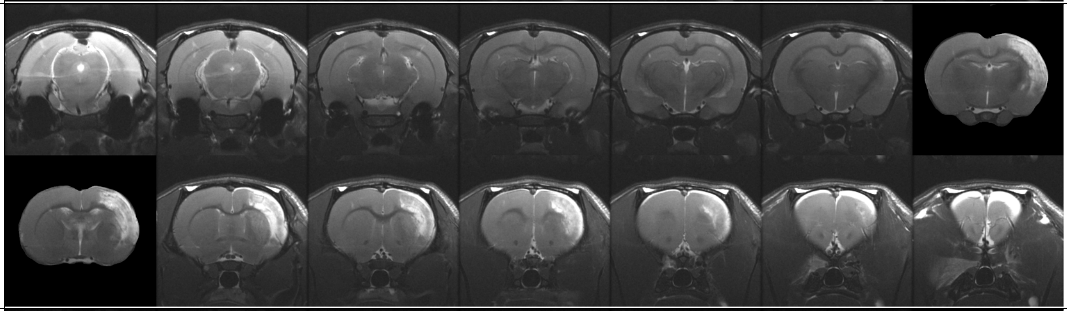
Day 30	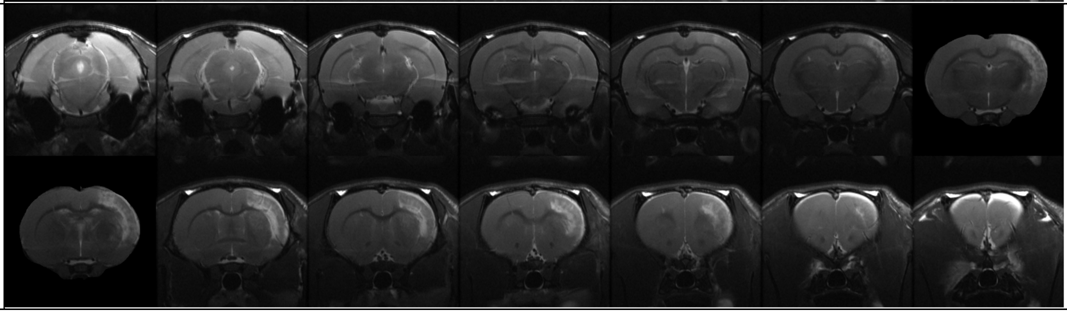

Table S4| **Raw data for Figure 2a**

Healthy	Saline			mPEG			hrGOT			mPEG–hrGOT			Angiopep–PEG–hrGOT		
0h	197	124	201	241	248	207	201	219	207	115	248	241	219	219	263
1h	197	228	193	197	269	227	4503	4768	4014	7180	5980	6149	4669	4954	6116
2h	241	207	199	285	269	227	3757	3606	3963	5344	7015	6499	5853	5283	5853
4h	263	207	221	219	269	227	1960	2937	3042	4350	4997	4888	5897	6532	4801
6h	285	207	243	241	207	207	1329	1644	1852	3451	5339	4549	5261	5919	5020
1d	263	248	263	219	228	186	229	373	372	2600	5121	4198	6160	6138	4735
2d	241	207	258	175	228	186	190	263	248	1510	2887	2400	3858	5261	2981
4d	219	228	261	175	207	249	197	263	228	870	1438	1458	3858	5064	2674
6d	197	166	239	175	207	249	205	219	207	440	921	866	1622	2302	1140
8d	241	186	241	175	248	207	203	241	197	335	590	548	965	701	877
14d	263	228	221	110	248	207	202	230	228	220	372	252	329	526	373
21d	203	187	203	187	156	187	172	179	148	179	172	187	263	416	285
30d	187	156	140	172	172	156	156	164	164	172	156	250	285	416	351

Table S5| **Raw data for Figure 2b**

MCAo	Saline			hrGOT				mPEG–hrGOT				Angiopep–PEG–hrGOT				
0h	254	535	329	359	333	285	263	241	241	351	263	351	329	285	263	263
1h	228	473	307	2359	2569	5086	5897	6313	6883	7365	4976	6795	4231	6006	5546	5305
6h	281	465	636	1070	1701	3135	3332	6927	6269	9360	5414	6752	7561	7278	7431	6905
1d	289	473	460	281	851	701	636	5677	6072	5984	6182	6225	4669	7058	6905	7146
2d	184	403	329	237	438	416	395	4077	4275	4910	4099	4264	5217	4538	4428	5414
4d	254	465	285	175	386	395	526	4033	3398	2828	3880	3244	3091	3463	3354	2389
6d	316	447	285	158	430	263	307	1885	2324	2477	2499	2521	2039	2367	2762	2367
8d	289	465	263	158	395	329	307	986	986	1249	2104	1381	2017	1556	1951	2674
14d	333	473	285	132	412	263	329	504	570	592	877	482	701	570	745	745
21d	368	482	241	132	421	241	307	351	482	438	592	307	329	395	351	460
30d	281	456	241	149	456	307	241	285	373	460	526	219	285	241	307	307

Table S6| **Raw data for Figure 3b**

	Saline					hrGOT					mPEG–hrGOT					Angiopep–PEG–hrGOT				
0d	32.6	34.6	40.9	42.3	43.7	44.2	40.0	43.6	43.8	44.0	44.2	46.2	43.8	30.6	46.9	41.7	48.3	47.1	40.8	38.7
1d	30.3	33.5	54.6	39.3	40.5	27.7	35.3	40.7	40.1	41.0	27.7	19.5	35.5	21.8	32.4	28.0	33.0	30.1	26.6	29.1
7d	19.5	25.2	34.0	33.2	22.1	16.8	28.5	17.2	32.1	17.0	16.8	8.1	14.3	9.1	21.6	16.6	17.2	16.0	16.6	12.3
14d	22.3	19.9	35.9	23.7	23.0	16.7	26.0	24.1	29.7	24.0	16.7	5.9	16.7	10.4	17.8	13.6	18.6	16.8	14.9	13.0
21d	17.9	20.4	27.0	23.7	23.0	18.1	26.0	22.2	29.7	24.0	18.1	5.5	12.1	8.1	15.7	13.2	19.4	14.9	12.7	13.1
30d	18.2	15.2	28.5	23.7	23.0	17.0	26.0	23.3	29.7	24.0	17.0	6.0	13.2	12.6	18.8	14.1	20.9	16.5	14.3	12.1

Table S7| **Raw data for Figure 4a**

	Saline			hrGOT				mPEG–hrGOT					Angiopep–PEG–hrGOT			
0d	100	100	100	100	100	100	100	100	100	100	100	100	100	100	100	100
3d	39	50	60	87	75	46	75	85	125	63	91	74	95	88	57	57
7d	46	45	59	59	53	58	71	90	104	75	100	69	113	92	76	67
14d	59	45	59	86	46	61	79	91	99	73	90	52	87	59	68	71
21d	47	46	66	91	79	59	69	82	91	52	55	55	66	56	42	62
30d	61	44	68	76	48	38	63	76	65	65	42	51	39	36	35	58

Table S8| **Raw data for Figure 4b**

	Saline			hrGOT				mPEG–hrGOT					Angiopep–PEG–hrGOT				
0d	50	71	50	50	44	44	50	53	50	50	50	47	50	50	50	50	50
3d	40	38	0	29	42	25	25	22	44	54	60	46	38	38	30	44	44
7d	0	13	10	33	50	40	27	42	47	43	50	46	47	33	36	55	55
14d	0	33	0	44	43	38	9	44	57	47	36	43	36	44	50	0	0
21d	0	18	20	47	43	38	46	56	53	47	46	47	38	53	43	50	50
30d	0	41	0	33	0	50	55	60	44	47	38	47	54	56	0	50	50

Table S9| **Raw data for Figure 5**

	Saline							hrGOT						mPEG–hrGOT			
0h	7	15	11	10	8	11	5	7	13	4	13	2	12	12	11	36	22
2h	7	9	11	20	8	5	2	20	59	18	20	21	26	91	19	18	75
4h	11	12	12	24		8	2	18	27	11	12	16		17	55	27	53
24h	8	9	9	4	19			2	4	9	3	8		18	20	20	

Multiple pairwise means comparison tables for Figures 2–5, and S4–5

Figure 2|

a)

Saline	MeanDiff	q Value	Prob	Alpha	Sig	LCL	UCL
**Hour 1 Hour 0**	56,15358	3,39873	0,40727	0,05	0	-27,37383	139,68098
**Hour 6 Hour 0**	70,76731	4,28324	0,14741	0,05	0	-12,7601	154,29471
**Day 1 Hour 0**	83,83755	5,07432	0,04863	0,05	1	0,31014	167,36495
**Day 2 Hour 0**	60,98429	3,69111	0,30107	0,05	0	-22,54311	144,5117
**Day 4 Hour 0**	61,60144	3,72847	0,2889	0,05	0	-21,92597	145,12884
**Day 6 Hour 0**	26,29508	1,59153	0,98448	0,05	0	-57,23232	109,82249
**Day 8 Hour 0**	48,53119	2,93738	0,60319	0,05	0	-34,99621	132,0586
**Day 14 Hour 0**	63,05112	3,81621	0,26165	0,05	0	-20,47629	146,57852
**Day 21 Hour 0**	23,28307	1,40922	0,99365	0,05	0	-60,24433	106,81048
**Day 30 Hour 0**	-13,11338	0,7937	0,99995	0,05	0	-96,64079	70,41402

PEG	MeanDiff	q Value	Prob	Alpha	Sig	LCL	UCL
**Hour 1 Hour 0**	6,48822	0,3353	1	0,05	0	-91,34013	104,31657
**Hour 6 Hour 0**	-13,70128	0,70805	0,99998	0,05	0	-111,52963	84,12707
**Day 1 Hour 0**	-21,10195	1,0905	0,9992	0,05	0	-118,9303	76,7264
**Day 2 Hour 0**	-35,71568	1,84571	0,95812	0,05	0	-133,54403	62,11267
**Day 4 Hour 0**	-21,73298	1,12311	0,99897	0,05	0	-119,56133	76,09537
**Day 6 Hour 0**	-21,73298	1,12311	0,99897	0,05	0	-119,56133	76,09537
**Day 8 Hour 0**	-21,92059	1,13281	0,99889	0,05	0	-119,74895	75,90776
**Day 14 Hour 0**	-43,84119	2,26561	0,86538	0,05	0	-141,66954	53,98716
**Day 21 Hour 0**	-55,24481	2,85493	0,63933	0,05	0	-153,07316	42,58354
**Day 30 Hour 0**	-65,6438	3,39232	0,4098	0,05	0	-163,47215	32,18455

hrGOT	MeanDiff	q Value	Prob	Alpha	Sig	LCL	UCL
**Hour 1 Hour 0**	2437,36616	21,19868	1,66158E-07	0,05	1	1856,09292	3018,63941
**Hour 6 Hour 0**	1399,33068	12,1705	7,76844E-07	0,05	1	818,05744	1980,60393
**Day 1 Hour 0**	115,88847	1,00793	0,99959	0,05	0	-465,38478	697,16171
**Day 2 Hour 0**	24,82156	0,21588	1	0,05	0	-556,45168	606,09481
**Day 4 Hour 0**	20,14624	0,17522	1	0,05	0	-561,127	601,41949
**Day 6 Hour 0**	1,41275	0,01229	1	0,05	0	-579,8605	582,686
**Day 8 Hour 0**	4,71529	0,04101	1	0,05	0	-576,55796	585,98854
**Day 14 Hour 0**	10,9915	0,0956	1	0,05	0	-570,28174	592,26475
**Day 21 Hour 0**	-42,58083	0,37034	1	0,05	0	-623,85408	538,69242
**Day 30 Hour 0**	-47,78032	0,41556	1	0,05	0	-629,05357	533,49292

mPEG–hrGOT	MeanDiff	q Value	Prob	Alpha	Sig	LCL	UCL
**Hour 1 Hour 0**	4543,74669	14,26801	8,67967E-08	0,05	1	2933,77192	6153,72145
**Hour 6 Hour 0**	4244,75044	13,32912	2,11914E-07	0,05	1	2634,77567	5854,7252
**Day 1 Hour 0**	3771,80061	11,84399	1,24604E-06	0,05	1	2161,82584	5381,77538
**Day 2 Hour 0**	2064,42008	6,48257	0,00536	0,05	1	454,44531	3674,39485
**Day 4 Hour 0**	1053,88848	3,30936	0,4432	0,05	0	-556,08629	2663,86325
**Day 6 Hour 0**	540,76335	1,69807	0,9757	0,05	0	-1069,21142	2150,73812
**Day 8 Hour 0**	289,39951	0,90876	0,99984	0,05	0	-1320,57526	1899,37428
**Day 14 Hour 0**	80,09232	0,2515	1	0,05	0	-1529,88245	1690,06709
**Day 21 Hour 0**	-22,00379	0,0691	1	0,05	0	-1631,97856	1587,97098
**Day 30 Hour 0**	-9,00506	0,02828	1	0,05	0	-1618,97982	1600,96971

Angiopep–mPEG–hrGOT	MeanDiff	q Value	Prob	Alpha	Sig	LCL	UCL
**Hour 1 Hour 0**	5509,37617	14,47423	8,03629E-08	0,05	1	3585,06516	7433,68718
**Hour 6 Hour 0**	5165,95352	13,57199	1,7567E-07	0,05	1	3241,6425	7090,26453
**Day 1 Hour 0**	5443,61438	14,30146	8,55398E-08	0,05	1	3519,30337	7367,9254
**Day 2 Hour 0**	3799,56977	9,98223	1,98539E-05	0,05	1	1875,25876	5723,88078
**Day 4 Hour 0**	3631,51188	9,54071	3,92491E-05	0,05	1	1707,20087	5555,82289
**Day 6 Hour 0**	1454,06612	3,82012	0,26048	0,05	0	-470,24489	3378,37714
**Day 8 Hour 0**	613,77666	1,61251	0,98298	0,05	0	-1310,53436	2538,08767
**Day 14 Hour 0**	175,36476	0,46072	1	0,05	0	-1748,94625	2099,67577
**Day 21 Hour 0**	87,68238	0,23036	1	0,05	0	-1836,62863	2011,99339
**Day 30 Hour 0**	116,90984	0,30715	1	0,05	0	-1807,40117	2041,22085

b)

Saline	MeanDiff	q Value	Prob	Alpha	Sig	LCL	UCL
**Hour 1 Hour 0**	-36,53432	0,52316	1	0,05	0	-389,58274	316,51409
**Hour 6 Hour 0**	87,68238	1,25559	0,99742	0,05	0	-265,36604	440,73079
**Day 1 Hour 0**	35,07295	0,50223	1	0,05	0	-317,97546	388,12137
**Day 2 Hour 0**	-67,22316	0,96262	0,99973	0,05	0	-420,27157	285,82526
**Day 4 Hour 0**	-37,9957	0,54409	1	0,05	0	-391,04411	315,05272
**Day 6 Hour 0**	-23,38197	0,33482	1	0,05	0	-376,43038	329,66645
**Day 8 Hour 0**	-33,61158	0,48131	1	0,05	0	-386,65999	319,43684
**Day 14 Hour 0**	-8,76824	0,12556	1	0,05	0	-361,81665	344,28018
**Day 21 Hour 0**	-8,76824	0,12556	1	0,05	0	-361,81665	344,28018
**Day 30 Hour 0**	-46,76394	0,66965	0,99999	0,05	0	-399,81235	306,28448

hrGOT	MeanDiff	q Value	Prob	Alpha	Sig	LCL	UCL
**Hour 1 Hour 0**	3667,3155	11,416	2,3E-07	0,05	1	2098,7014	5235,9296
**Hour 6 Hour 0**	1999,1583	6,22316	0,00438	0,05	1	430,54416	3567,7723

**Day 1 Hour 0**	306,88833	0,95531	0,99979	0,05	0	-1261,7258	1875,5024
**Day 2 Hour 0**	61,37767	0,19106	1	0,05	0	-1507,2364	1629,9918
**Day 4 Hour 0**	60,28164	0,18765	1	0,05	0	-1508,3325	1628,8957
**Day 6 Hour 0**	-20,82457	0,06482	1	0,05	0	-1589,4387	1547,7895
**Day 8 Hour 0**	-13,15236	0,04094	1	0,05	0	-1581,7664	1555,4617
**Day 14 Hour 0**	-26,30471	0,08188	1	0,05	0	-1594,9188	1542,3094
**Day 21 Hour 0**	-35,07295	0,10918	1	0,05	0	-1603,687	1533,5411
**Day 30 Hour 0**	-21,92059	0,06824	1	0,05	0	-1590,5347	1546,6935

mPEG–hrGOT	MeanDiff	q Value	Prob	Alpha	Sig	LCL	UCL
**Hour 1 Hour 0**	6110,3658	18,4075	2,8E-08	0,05	1	4489,4772	7731,2544
**Hour 6 Hour 0**	6718,6623	20,24	1,3E-07	0,05	1	5097,7737	8339,5509
**Day 1 Hour 0**	5704,8348	17,1858	0	0,05	1	4083,9462	7325,7234
**Day 2 Hour 0**	4066,2703	12,2496	9,9E-08	0,05	1	2445,3817	5687,159
**Day 4 Hour 0**	3260,6885	9,82281	3,1E-06	0,05	1	1639,7999	4881,5771
**Day 6 Hour 0**	2022,1749	6,0918	0,00563	0,05	1	401,28625	3643,0635
**Day 8 Hour 0**	1057,6687	3,18622	0,48653	0,05	0	-563,21993	2678,5573
**Day 14 Hour 0**	361,68981	1,08959	0,99933	0,05	0	-1259,1988	1982,5784
**Day 21 Hour 0**	191,8052	0,57781	1	0,05	0	-1429,0834	1812,6938
**Day 30 Hour 0**	137,00372	0,41272	1	0,05	0	-1483,8849	1757,8923

Angiopep–mPEG–hrGOT	MeanDiff	q Value	Prob	Alpha	Sig	LCL	UCL
**Hour 1 Hour 0**	5337,6648	20,566	1,7E-07	0,05	1	4064,6496	6610,6801
**Hour 6 Hour 0**	6846,5325	24,4228	1,5E-08	0,05	1	5471,5188	8221,5461
**Day 1 Hour 0**	5907,6003	22,7619	4,6E-08	0,05	1	4634,585	7180,6156
**Day 2 Hour 0**	4420,6533	15,7693	3,1E-09	0,05	1	3045,6396	5795,667
**Day 4 Hour 0**	2981,2009	11,4866	2,9E-07	0,05	1	1708,1856	4254,2162
**Day 6 Hour 0**	2115,3374	8,15038	0,00011	0,05	1	842,32211	3388,3527
**Day 8 Hour 0**	1419,3585	5,46878	0,01907	0,05	1	146,34322	2692,3738
**Day 14 Hour 0**	317,84863	1,22467	0,99816	0,05	0	-955,16667	1590,8639
**Day 21 Hour 0**	38,36104	0,1478	1	0,05	0	-1234,6543	1311,3763
**Day 30 Hour 0**	-43,84119	0,16892	1	0,05	0	-1316,8565	1229,1741

Figure 3|

0 Day	Index	DF	|t|value	Prob>|t|	Alpha	Sig Flag	95.00% LCL	95.00% UCL
**Saline hrGOT**	0	12	1,39	1,00E+00	0,05	0	-17,73	45,80
**Saline mPEG–hrGOT**	1	12	0,06	1,00E+00	0,05	0	-31,15	32,38
**Saline Angiopep–PEG–hrGOT**	2	12	0,34	1,00E+00	0,05	0	-28,36	35,18

1 Day	Index	DF	|t|value	Prob>|t|	Alpha	Sig Flag	95.00% LCL	95.00% UCL
Saline hrGOT	0	12	2,11	3,37E-01	0,05	0	-7,89	39,96
Saline mPEG–hrGOT	1	12	4,81	2,56E-03	0,05	1	12,58	60,42
Saline Angiopep–PEG–hrGOT	2	12	4,46	4,65E-03	0,05	1	9,94	57,78

7 Day	Index	DF	|t|value	Prob>|t|	Alpha	Sig Flag	95.00% LCL	95.00% UCL
Saline vs hrGOT	0	12	2,00	4,15E-01	0,05	0	-9,74	43,37
Saline mPEG–hrGOT	1	12	4,30	6,22E-03	0,05	1	9,64	62,75
Saline Angiopep–PEG–hrGOT	2	12	3,87	1,34E-02	0,05	1	6,02	59,13

14 Day	Index	DF	|t|value	Prob>|t|	Alpha	Sig Flag	95.00% LCL	95.00% UCL
Saline vs hrGOT	0	12	1,18	1,00E+00	0,05	0	-14,18	31,05
Saline mPEG–hrGOT	1	12	4,51	4,25E-03	0,05	1	9,77	55,00
Saline Angiopep–PEG–hrGOT	2	12	4,06	9,52E-03	0,05	1	6,50	51,73

21 Day	Index	DF	|t|value	Prob>|t|	Alpha	Sig Flag	95.00% LCL	95.00% UCL
Saline vs hrGOT	0	12	0,32	1,00E+00	0,05	0	-16,94	20,80
Saline mPEG–hrGOT	1	12	4,96	1,99E-03	0,05	1	10,81	48,55
Saline Angiopep–PEG–hrGOT	2	12	4,01	1,04E-02	0,05	1	5,13	42,88

30 Day	Index	DF	|t|value	Prob>|t|	Alpha	Sig Flag	95.00% LCL	95.00% UCL
Saline vs hrGOT	0	12	0,03	1,00E+00	0,05	0	-20,90	20,54
Saline mPEG–hrGOT	1	12	3,51	2,59E-02	0,05	1	2,33	43,77
Saline Angiopep–PEG–hrGOT	2	12	3,04	6,12E-02	0,05	0	-0,72	40,72

Figure 4|

a)

Saline	Index	DF	|t|value	Prob>|t|	Alpha	Sig Flag	95.00% LCL	95.00% UCL
Day0 Day3	0	10	9,31847	4,54E-05	0,05	1	29,59699	70,85538
Day0 Day7	1	10	9,30569	4,59E-05	0,05	1	29,52812	70,78652
Day0 Day14	2	10	8,42221	1,12E-04	0,05	1	24,76618	66,02458
Day0 Day21	3	10	8,7457	8,03E-05	0,05	1	26,5098	67,7682
Day0 Day30	4	10	7,84352	2,10E-04	0,05	1	21,64709	62,90548

hrGOT	Index	DF	|t|value	Prob>|t|	Alpha	Sig Flag	95.00% LCL	95.00% UCL

**Day0 Day3**	0	15	3,9166	2,06E-02	0,05	1	3,20659	54,81271
**Day0 Day7**	1	15	5,38987	1,13E-03	0,05	1	14,1189	65,72502
**Day0 Day14**	2	15	4,36384	8,34E-03	0,05	1	6,51925	58,12537
**Day0 Day21**	3	15	3,44521	5,41E-02	0,05	0	-0,28495	51,32117
**Day0 Day30**	4	15	5,87563	4,57E-04	0,05	1	17,71686	69,32298

**mPEG–hrGOT**	**Index**	**DF**	**|t|value**	**Prob>|t|**	**Alpha**	**Sig Flag**	**95.00% LCL**	**95.00% UCL**
**Day0 Day3**	0	20	1,57021	1,00E+00	0,05	0	-17,19733	47,87552
**Day0 Day7**	1	20	1,5761	1,00E+00	0,05	0	-17,13982	47,93302
**Day0 Day14**	2	20	2,24214	5,47E-01	0,05	0	-10,6334	54,43945
**Day0 Day21**	3	20	3,67346	2,26E-02	0,05	1	3,34889	68,42174
**Day0 Day30**	4	20	4,40343	4,11E-03	0,05	1	10,47987	75,55271

**Angiopep–mPEG–hrGOT**	**Index**	**DF**	**|t|value**	**Prob>|t|**	**Alpha**	**Sig Flag**	**95.00% LCL**	**95.00% UCL**
**Day0 Day3**	0	15	2,98164	1,40E-01	0,05	0	-4,3557	56,09299
**Day0 Day7**	1	15	1,48656	1,00E+00	0,05	0	-17,32699	43,12171
**Day0 Day14**	2	15	3,33533	6,78E-02	0,05	0	-1,28709	59,16161
**Day0 Day21**	3	15	5,03514	2,22E-03	0,05	1	13,46046	73,90916
**Day0 Day30**	4	15	6,69701	1,07E-04	0,05	1	27,87888	88,32757

b)

**Saline**	**Index**	**DF**	**|t|value**	**Prob>|t|**	**Alpha**	**Sig Flag**	**95.00% LCL**	**95.00% UCL**
**Day0 Day3**	0	10	2,9828	2,06E-01	0,05	0	-8,8649	71,48395
**Day0 Day7**	1	10	4,72938	1,21E-02	0,05	1	9,46843	89,81729
**Day0 Day14**	2	10	4,38535	2,05E-02	0,05	1	5,85732	86,20617
**Day0 Day21**	3	10	4,23138	2,61E-02	0,05	1	4,24116	84,59001
**Day0 Day30**	4	10	4,13629	3,04E-02	0,05	1	3,24294	83,5918

**hrGOT**	**Index**	**DF**	**|t|value**	**Prob>|t|**	**Alpha**	**Sig Flag**	**95.00% LCL**	**95.00% UCL**
**Day0 Day3**	0	15	1,66393	1,00E+00	0,05	0	-18,57998	52,55815
**Day0 Day7**	1	15	0,92036	1,00E+00	0,05	0	-26,17197	44,96616
**Day0 Day14**	2	15	1,32306	1,00E+00	0,05	0	-22,06035	49,07778
**Day0 Day21**	3	15	0,34415	1,00E+00	0,05	0	-32,05525	39,08288
**Day0 Day30**	4	15	1,23199	1,00E+00	0,05	0	-22,99015	48,14798

**mPEG–hrGOT**	**Index**	**DF**	**|t|value**	**Prob>|t|**	**Alpha**	**Sig Flag**	**95.00% LCL**	**95.00% UCL**

**Day0 Day3**	0	20	0,90717	1,00E+00	0,05	0	-12,83792	22,44903
**Day0 Day7**	1	20	0,84056	1,00E+00	0,05	0	-13,19077	22,09618
**Day0 Day14**	2	20	0,82394	1,00E+00	0,05	0	-13,27878	22,00818
**Day0 Day21**	3	20	0,03482	1,00E+00	0,05	0	-17,45904	17,82791
**Day0 Day30**	4	20	0,54103	1,00E+00	0,05	0	-14,77746	20,50949

**Angiopep–mPEG–hrGOT**	**Index**	**DF**	**|t|value**	**Prob>|t|**	**Alpha**	**Sig Flag**	**95.00% LCL**	**95.00% UCL**
**Day0 Day3**	0	20	1,1341	1,00E+00	0,05	0	-21,73525	44,1797
**Day0 Day7**	1	20	0,50923	1,00E+00	0,05	0	-27,91851	37,99644
**Day0 Day14**	2	20	2,42311	3,75E-01	0,05	0	-8,9802	56,93475
**Day0 Day21**	3	20	0,33757	1,00E+00	0,05	0	-29,61714	36,29781
**Day0 Day30**	4	20	0,80653	1,00E+00	0,05	0	-24,97671	40,93824

Figure 5|

**Saline**	**MeanDiff**	**q Value**	**Prob**	**Alpha**	**Sig**	**LCL**	**UCL**
**Hour 2 Hour 0**	14,50999	2,50576	0,33223	0,05	0	-9,80297	38,82294
**Hour 4 Hour 0**	-3,5272	0,60912	0,97205	0,05	0	-27,84016	20,78575
**Day 1 Hour 0**	-9,3804	1,61992	0,67006	0,05	0	-33,69335	14,93256

**hrGOT**	**MeanDiff**	**q Value**	**Prob**	**Alpha**	**Sig**	**LCL**	**UCL**
**Hour 2 Hour 0**	21,02867	4,85251	0,03628	0,05	1	1,40279	40,65454
**Hour 4 Hour 0**	8,0415	1,85563	0,581	0,05	0	-11,58438	27,66738
**Day 1 Hour 0**	-3,65	0,84226	0,93061	0,05	0	-23,27588	15,97588

**mPEG–hrGOT**	**MeanDiff**	**q Value**	**Prob**	**Alpha**	**Sig**	**LCL**	**UCL**
**Hour 2 Hour 0**	30,73615	2,68371	0,28344	0,05	0	-18,00881	79,48111
**Hour 4 Hour 0**	17,73852	1,54883	0,69956	0,05	0	-31,00644	66,48348
**Day 1 Hour 0**	-0,99226	0,08021	0,99993	0,05	0	-53,64284	51,65831

Figure S4|

a)

**Saline**	**MeanDiff**	**q Value**	**Prob**	**Alpha**	**Sig**	**LCL**	**UCL**
**Hour 1 Hour 0**	2,46124	0,15978	1	0,05	0	-75,80054	80,72302
**Hour 6 Hour 0**	6,3746	0,41383	1	0,05	0	-71,88718	84,63638
**Day 1 Hour 0**	8,9671	0,52067	1	0,05	0	-78,53223	96,46643
**Day 2 Hour 0**	11,52679	0,7483	0,99997	0,05	0	-66,73499	89,78857
**Day 4 Hour 0**	22,63516	1,46944	0,99115	0,05	0	-55,62662	100,89694

**Day 6 Hour 0**	12,19132	0,79144	0,99995	0,05	0	-66,07046	90,4531
**Day 8 Hour 0**	-1,65723	0,10758	1	0,05	0	-79,91901	76,60455
**Day 14 Hour 0**	3,09295	0,20079	1	0,05	0	-75,16883	81,35473
**Day 21 Hour 0**	-4,90606	0,31849	1	0,05	0	-83,16784	73,35572
**Day 30 Hour 0**	50,11076	3,25311	0,46778	0,05	0	-28,15102	128,37254

PEG	MeanDiff	q Value	Prob	Alpha	Sig	LCL	UCL
**Hour 1 Hour 0**	12,31438	0,88786	0,99986	0,05	0	-58,1522	82,78096
**Hour 6 Hour 0**	11,70728	0,84409	0,99991	0,05	0	-58,7593	82,17386
**Day 1 Hour 0**	35,69612	2,57368	0,75691	0,05	0	-34,77046	106,1627
**Day 2 Hour 0**	19,78013	1,42614	0,99292	0,05	0	-50,68645	90,24671
**Day 4 Hour 0**	27,64788	1,9934	0,93252	0,05	0	-42,8187	98,11446
**Day 6 Hour 0**	8,45845	0,60985	0,99999	0,05	0	-62,00813	78,92503
**Day 8 Hour 0**	-20,49799	1,32187	0,99605	0,05	0	-99,28202	58,28604
**Day 14 Hour 0**	-1,69825	0,12244	1	0,05	0	-72,16483	68,76833
**Day 21 Hour 0**	-19,61605	1,41431	0,99335	0,05	0	-90,08263	50,85053
**Day 30 Hour 0**	-3,42112	0,24666	1	0,05	0	-73,8877	67,04546

hrGOT	MeanDiff	q Value	Prob	Alpha	Sig	LCL	UCL
**Hour 1 Hour 0**	5,49676	0,42594	1	0,05	0	-61,7656	72,75912
**Hour 6 Hour 0**	26,96694	2,08965	0,90861	0,05	0	-40,29542	94,2293
**Day 1 Hour 0**	24,78464	1,92054	0,94331	0,05	0	-42,47772	92,047
**Day 2 Hour 0**	16,94971	1,31342	0,99586	0,05	0	-50,31265	84,21207
**Day 4 Hour 0**	18,84486	1,46027	0,99081	0,05	0	-48,4175	86,10722
**Day 6 Hour 0**	19,7268	1,36724	0,99437	0,05	0	-55,4748	94,92841
**Day 8 Hour 0**	10,97711	0,76081	0,99996	0,05	0	-64,22449	86,17871
**Day 14 Hour 0**	19,75142	1,36894	0,99432	0,05	0	-55,45019	94,95302
**Day 21 Hour 0**	20,19444	1,39965	0,99328	0,05	0	-55,00717	95,39604
**Day 30 Hour 0**	30,4824	2,11269	0,90307	0,05	0	-44,7192	105,68401

mPEG–hrGOT	MeanDiff	q Value	Prob	Alpha	Sig	LCL	UCL
**Hour 1 Hour 0**	13,30708	0,91173	0,99982	0,05	0	-61,69946	88,31362
**Hour 6 Hour 0**	25,72812	1,76276	0,9676	0,05	0	-49,27843	100,73466
**Day 1 Hour 0**	16,96612	1,16243	0,99852	0,05	0	-58,04043	91,97266
**Day 2 Hour 0**	23,69349	1,62335	0,98131	0,05	0	-51,31305	98,70004
**Day 4 Hour 0**	8,58971	0,58852	1	0,05	0	-66,41683	83,59626
**Day 6 Hour 0**	-13,77471	0,94377	0,99975	0,05	0	-88,78126	61,23183
**Day 8 Hour 0**	-7,63393	0,46782	1	0,05	0	-91,4938	76,22593
**Day 14 Hour 0**	9,59061	0,6571	0,99999	0,05	0	-65,41593	84,59716
**Day 21 Hour 0**	3,47855	0,21317	1	0,05	0	-80,38132	87,33841

**Day 30 Hour 0**	24,94052	1,52839	0,98777	0,05	0	-58,91935	108,80039

Angiopep–PEG–hrGOT	MeanDiff	q Value	Prob	Alpha	Sig	LCL	UCL
**Hour 1 Hour 0**	33,73123	3,85539	0,25009	0,05	0	-10,50034	77,96281
**Hour 6 Hour 0**	18,31159	2,09297	0,91162	0,05	0	-25,91998	62,54317
**Day 1 Hour 0**	45,69284	5,22257	0,03896	0,05	1	1,46126	89,92441
**Day 2 Hour 0**	8,86045	1,01273	0,99957	0,05	0	-35,37113	53,09202
**Day 4 Hour 0**	48,92936	5,59249	0,0221	0,05	1	4,69779	93,16094
**Day 6 Hour 0**	13,56141	1,55003	0,98714	0,05	0	-30,67017	57,79298
**Day 8 Hour 0**	30,2732	3,46014	0,38344	0,05	0	-13,95838	74,50477
**Day 14 Hour 0**	60,49717	6,91466	0,00266	0,05	1	16,2656	104,72874
**Day 21 Hour 0**	32,32833	3,69504	0,29977	0,05	0	-11,90324	76,5599
**Day 30 Hour 0**	52,47354	5,99758	0,01168	0,05	1	8,24197	96,70511

b)

Saline	MeanDiff	q Value	Prob	Alpha	Sig	LCL	UCL
**Hour 1 Hour 0**	-69,25789	4,25499	0,15292	0,05	0	-151,54648	13,03069
**Hour 6 Hour 0**	-56,10825	3,44711	0,38844	0,05	0	-138,39683	26,18034
**Day 1 Hour 0**	-17,98045	1,10466	0,9991	0,05	0	-100,26904	64,30813
**Day 2 Hour 0**	-60,71215	3,72996	0,28842	0,05	0	-143,00073	21,57644
**Day 4 Hour 0**	-55,47959	3,40849	0,40344	0,05	0	-137,76818	26,80899
**Day 6 Hour 0**	-55,98252	3,43939	0,39141	0,05	0	-138,2711	26,30607
**Day 8 Hour 0**	-22,382	1,37508	0,99473	0,05	0	-104,67059	59,90658
**Day 14 Hour 0**	-57,64353	3,54144	0,35309	0,05	0	-139,93212	24,64505
**Day 21 Hour 0**	-27,28059	1,67603	0,97777	0,05	0	-109,56917	55,008
**Day 30 Hour 0**	-70,58101	4,33627	0,1375	0,05	0	-152,8696	11,70757

hrGOT	MeanDiff	q Value	Prob	Alpha	Sig	LCL	UCL
**Hour 1 Hour 0**	-26,27671	1,82565	0,96092	0,05	0	-99,04169	46,48827
**Hour 6 Hour 0**	-46,94956	3,26195	0,46279	0,05	0	-119,71454	25,81542
**Day 1 Hour 0**	8,86892	0,61619	0,99999	0,05	0	-63,89606	81,6339
**Day 2 Hour 0**	-25,72467	1,78729	0,9659	0,05	0	-98,48965	47,04031
**Day 4 Hour 0**	-48,61549	3,3777	0,4156	0,05	0	-121,38047	24,14949
**Day 6 Hour 0**	-39,85855	2,76928	0,67642	0,05	0	-112,62353	32,90643
**Day 8 Hour 0**	-41,00093	2,84865	0,64207	0,05	0	-113,76591	31,76405
**Day 14 Hour 0**	4,72668	0,3284	1	0,05	0	-68,0383	77,49166
**Day 21 Hour 0**	-49,85512	3,46382	0,38204	0,05	0	-122,6201	22,90987
**Day 30 Hour 0**	16,89996	1,17417	0,99851	0,05	0	-55,86502	89,66494

mPEG–hrGOT	MeanDiff	q Value	Prob	Alpha	Sig	LCL	UCL
Hour 1 Hour 0	-12,9807	0,99543	0,99969	0,05	0	-76,65527	50,69387
Hour 6 Hour 0	-11,77545	0,90301	0,99987	0,05	0	-75,45002	51,89912
Day 1 Hour 0	5,85089	0,44868	1	0,05	0	-57,82368	69,52546
Day 2 Hour 0	-10,85752	0,83262	0,99994	0,05	0	-74,53209	52,81705
Day 4 Hour 0	-0,45823	0,03514	1	0,05	0	-64,1328	63,21634
Day 6 Hour 0	-23,07352	1,76941	0,97079	0,05	0	-86,74809	40,60105
Day 8 Hour 0	-22,04803	1,69077	0,97861	0,05	0	-85,7226	41,62654
Day 14 Hour 0	5,90025	0,45246	1	0,05	0	-57,77432	69,57482
Day 21 Hour 0	19,48062	1,49388	0,9913	0,05	0	-44,19395	83,15519
Day 30 Hour 0	10,75438	0,82471	0,99994	0,05	0	-52,92019	74,42895

Angiopep–mPEG–hrGOT	MeanDiff	q Value	Prob	Alpha	Sig	LCL	UCL
Hour 1 Hour 0	-33,44924	1,72263	0,97325	0,05	0	-131,61585	64,71737
Hour 6 Hour 0	18,6317	0,95953	0,99973	0,05	0	-79,53491	116,79831
Day 1 Hour 0	35,92358	1,85005	0,95749	0,05	0	-62,24303	134,09019
Day 2 Hour 0	7,55562	0,38911	1	0,05	0	-90,61099	105,72223
Day 4 Hour 0	5,79638	0,29851	1	0,05	0	-92,37024	103,96299
Day 6 Hour 0	39,46957	2,03267	0,92508	0,05	0	-58,69704	137,63619
Day 8 Hour 0	-5,94077	0,30595	1	0,05	0	-104,10738	92,22584
Day 14 Hour 0	18,43623	0,94946	0,99976	0,05	0	-79,73039	116,60284
Day 21 Hour 0	-5,25907	0,27084	1	0,05	0	-103,42569	92,90754
Day 30 Hour 0	19,65031	1,01198	0,99958	0,05	0	-78,5163	117,81692

Figure S5|

Saline	MeanDiff	q Value	Prob	Alpha	Sig	LCL	UCL
Hour 2 Hour 0	-0.60225	0.37835	0.9927	0.05	0	-8.05377	6.84928
Hour 4 Hour 0	2.67146	1.87639	0.5763	0.05	0	-3.99339	9.33631
Hour 4 Hour 2	3.27371	2.05665	0.50864	0.05	0	-4.17782	10.72524
Day 1 Hour 0	1.23135	0.86488	0.9252	0.05	0	-5.4335	7.8962
Day 1 Hour 2	1.8336	1.15192	0.84596	0.05	0	-5.61793	9.28513
Day 1 Hour 4	-1.44011	1.01151	0.88803	0.05	0	-8.10496	5.22474

hrGOT	MeanDiff	q Value	Prob	Alpha	Sig	LCL	UCL
Hour 2 Hour 0	2.4196	1.37133	0.77033	0.05	0	-5.84011	10.6793
Hour 4 Hour 0	1.61765	1.02504	0.88423	0.05	0	-5.77006	9.00535
Hour 4 Hour 2	-0.80195	0.45451	0.98755	0.05	0	-9.06166	7.45776
Day 1 Hour 0	3.0373	1.92461	0.5579	0.05	0	-4.35041	10.425
Day 1 Hour 2	0.6177	0.35009	0.99418	0.05	0	-7.642	8.87741
Day 1 Hour 4	1.41965	0.89957	0.91708	0.05	0	-5.96805	8.80736

mPEG–hrGOT	MeanDiff	q Value	Prob	Alpha	Sig	LCL	UCL
**Hour 2 Hour 0**	-0.57651	0.38719	0.99219	0.05	0	-7.54684	6.39381
**Hour 4 Hour 0**	1.3945	0.93655	0.90795	0.05	0	-5.57582	8.36483
**Hour 4 Hour 2**	1.97101	1.32374	0.78756	0.05	0	-4.99931	8.94134
**Day 1 Hour 0**	0.03783	0.02272	1	0.05	0	-7.75523	7.83089
**Day 1 Hour 2**	0.61434	0.36904	0.99321	0.05	0	-7.17872	8.4074
**Day 1 Hour 4**	-1.35667	0.81495	0.93611	0.05	0	-9.14973	6.43639
