# Supplementary material for: Sustained blood glutamate scavenging enhances protection in ischemic stroke
Source: Commun Biol. 2020 Dec 3;3:729. doi: 10.1038/s42003-020-01406-1 (PMC7713697; doi:10.1038/s42003-020-01406-1)
Supplement: Supplementary file 2 — Reporting Summary [file 42003_2020_1406_MOESM2_ESM.pdf]

nature research

Corresponding author(s): Marc A Gauthier

Last updated by author(s): Apr 6, 2020

Reporting Summary

Nature Research wishes to improve the reproducibility of the work that we publish. This form provides structure for consistency and transparency in reporting. For further information on Nature Research policies, see Authors & Referees and the Editorial Policy Checklist.

Statistics

For all statistical analyses, confirm that the following items are present in the figure legend, table legend, main text, or Methods section.

n/a	Confirmed	
[ ]	[x]	The exact sample size (n) for each experimental group/condition, given as a discrete number and unit of measurement
[x]	[ ]	A statement on whether measurements were taken from distinct samples or whether the same sample was measured repeatedly
[ ]	[x]	The statistical test(s) used AND whether they are one- or two-sided<br>*Only common tests should be described solely by name; describe more complex techniques in the Methods section.*
[x]	[ ]	A description of all covariates tested
[x]	[ ]	A description of any assumptions or corrections, such as tests of normality and adjustment for multiple comparisons
[ ]	[x]	A full description of the statistical parameters including central tendency (e.g. means) or other basic estimates (e.g. regression coefficient) AND variation (e.g. standard deviation) or associated estimates of uncertainty (e.g. confidence intervals)
[x]	[ ]	For null hypothesis testing, the test statistic (e.g. F, t, r) with confidence intervals, effect sizes, degrees of freedom and P value noted<br>*Give P values as exact values whenever suitable.*
[x]	[ ]	For Bayesian analysis, information on the choice of priors and Markov chain Monte Carlo settings
[x]	[ ]	For hierarchical and complex designs, identification of the appropriate level for tests and full reporting of outcomes
[x]	[ ]	Estimates of effect sizes (e.g. Cohen's d, Pearson's r), indicating how they were calculated

Our web collection on statistics for biologists contains articles on many of the points above.

Software and code

Policy information about availability of computer code

Data collection	No software was used
Data analysis	Microcal Origin 2019

For manuscripts utilizing custom algorithms or software that are central to the research but not yet described in published literature, software must be made available to editors/reviewers. We strongly encourage code deposition in a community repository (e.g. GitHub). See the Nature Research guidelines for submitting code & software for further information.

Data

Policy information about availability of data

All manuscripts must include a data availability statement. This statement should provide the following information, where applicable:

- Accession codes, unique identifiers, or web links for publicly available datasets
- A list of figures that have associated raw data
- A description of any restrictions on data availability

All relevant data is supplied in the manuscript or the SI

Field-specific reporting

Please select the one below that is the best fit for your research. If you are not sure, read the appropriate sections before making your selection.

[x] Life sciences [ ] Behavioural & social sciences [ ] Ecological, evolutionary & environmental sciences

For a reference copy of the document with all sections, see nature.com/documents/nr-reporting-summary-flat.pdf

Life sciences study design

All studies must disclose on these points even when the disclosure is negative.

Sample size	No sample size calculation was performed. Sample size was chosen based on prior experience yielding reasonable standard deviation.
Data exclusions	Exclusion criteria for inclusion for animals is described in the methods sections. Once included in the experiment, no data was excluded.
Replication	Values for control experiments are consistent with expectations from the literature.
Randomization	Randomization is not applicable in this work.
Blinding	The most subjective experiments were performed double-blinded, as described in the methods.

Reporting for specific materials, systems and methods

We require information from authors about some types of materials, experimental systems and methods used in many studies. Here, indicate whether each material, system or method listed is relevant to your study. If you are not sure if a list item applies to your research, read the appropriate section before selecting a response.

Materials & experimental systems

n/a	Involved in the study	
☒	☐	Antibodies
☒	☐	Eukaryotic cell lines
☒	☐	Palaeontology
☐	☒	Animals and other organisms
☒	☐	Human research participants
☒	☐	Clinical data

Methods

n/a	Involved in the study	
☒	☐	ChIP-seq
☒	☐	Flow cytometry
☐	☒	MRI-based neuroimaging

Animals and other organisms

Policy information about studies involving animals; ARRIVE guidelines recommended for reporting animal research

Laboratory animals	Rats, Sprague–Dawley, Male, 250–300 g (~7–8 weeks)
Wild animals	The study did not involve wild animals
Field-collected samples	The study did not involve samples collected from the field
Ethics oversight	Experimental protocols were approved by the local Animal Care Committee according to the requirements of the European Union (86/609/CEE, 2003/65/CE and 2010/63/EU)

Note that full information on the approval of the study protocol must also be provided in the manuscript.

Magnetic resonance imaging

Experimental design

Design type	- Middle cerebral artery occlusion (MCAo) status in each animal was evaluated in a non-invasive manner with the time-of-flight magnetic resonance angiography (TOF-MRA).<br>- Apparent diffusion coefficient (ADC) maps were acquired during MCAo from diffusion-weighted images (DWI) using a spin echo echo-planar imaging sequence (DTI-EPI) to calculate lesion volume during occlusion.<br>- The progression of ischemic lesions and infarct volumes were determined from T2-maps calculated from T2-weighted images.
Design specifications	Sequences time:<br>- Time-of-flight magnetic resonance angiography (TOF-MRA): 7 minutes.<br>- Diffusion-weighted images (DWI): 3 minutes.<br>- T2-weighted images (multi-slice-multi-echo (MSME)): 20 minutes.<br><br>Animal studies:<br>Basal animal study (during MCAo): TOF-MRA + DWI<br>Follow-ups (1, 7, 14, 21, and 30 days after the onset of ischemia): T2 sequence
Behavioral performance measures	- Variables: area, volume, gray value

Behavioral performance measures

- All data are expressed as mean±s.d
- The criterion for statistical significance was P<0.05

Acquisition

Imaging type(s)

Diffusion, perfusion and structural

Field strength

9.4 Tesla

Sequence & imaging parameters

- ADC maps were acquired from diffusion-weighted images (DWI) using a spin echo echo-planar imaging sequence (DTI-EPI) with the following acquisition parameters: echo time (ET)= 24.89 ms, repetition time (RT)= 4.5 s, spectral bandwidth (SW)= 200 KHz, 7 b-values of 0, 300, 600, 900, 1200, 1600, and 2000 s/mm2, flip angle (FA)= 90º, number of averages (NA) = 3, 14 consecutive slices of 1 mm, 24 × 16 mm2 FOV (with saturation bands to suppress signal outside this FOV), a matrix size of 96 × 64 (isotropic in-plane resolution of 250 µm/pixel × 250 µm/pixel) and implemented with fat suppression option.

- Time-of-flight magnetic resonance angiography (TOF-MRA) scan was performed with a 3D-Flash sequence with an echo time (ET)= 2.8 ms, repetition time (RT)=15 ms, flip angle (FA)= 30º, number of averages (NA) = 2, spectral bandwidth (SW)= 98 KHz, 1 slice of 14 mm, 30.72 × 30.72 × 14 mm3 FOV (with saturation bands to suppress signal outside this FOV), a matrix size of 256 × 256 × 58 (resolution of 120 µm/pixel × 120 µm/pixel × 241 µm/pixel) and implemented without fat suppression option.

- T2-maps calculated from T2-weighted images acquired using a MSME sequence: with an echo time (ET)= 9 ms, repetition time (RT)= 3 s, 16 echoes with 9 ms echo spacing, flip angle (FA) = 180º, number of averages (NA) = 2, spectral bandwidth (SW)= 75 KHz, 14 slices of 1 mm, 19.2 × 19.2 mm2 FOV (with saturation bands to suppress signal outside this FOV), a matrix size of 192 × 192 (isotropic in-plane resolution of 100 µm/pixel × 100 µm/pixel) and implemented without fat suppression option.

Area of acquisition

Region determined for each sequence:
- Diffusion 14 consecutive slices of 1 mm, 24 × 16 mm2 FOV;
- TOF-MRA 1 slice of 14 mm, 30.72 × 30.72 × 14 mm3 FOV;
- MSME 14 slices of 1 mm, 19.2 × 19.2 mm2 FOV

Diffusion MRI ☒ Used ☐ Not used

Parameters

b values of 0, 300, 600, 900, 1200, 1600 and 2000 s/mm2 applied in the z direction

Preprocessing

Preprocessing software

- ParaVision 6.0.1 (Bruker BioSpin, Ettligen, Germany)
- ImageJ (https://imagej.nih.gov/ij/)

Normalization

No

Normalization template

No

Noise and artifact removal

No

Volume censoring

- Infarct volumes were determined from ADC maps and T2 relaxation maps by manually selecting areas of reduced ADC values or hyperintense T2 signal by a researcher blinded to the animal protocols (Neurology. 1995;45(1):172-7; Dis Model Mech. 2017;10(12):1433-1438).

- Lesion volume as a percentage of the ipsilateral hemispheric volume was calculated as (lesion volume [mm3]/ ipsilateral hemispheric volume [mm3]) × 100.

Statistical modeling & inference

Model type and settings

No

Effect(s) tested

No

Specify type of analysis: ☐ Whole brain ☒ ROI-based ☐ Both

Anatomical location(s)

- Anatomical loctions were determined semi-automated.
- Lesion volumes were determined from ADC maps and T2 relaxation maps by manually selecting areas of reduced ADC values or hyperintense T2 signal by a researcher blinded to the animal protocols.

Statistic type for inference
(See Eklund et al. 2016)

No

Correction

No

Models & analysis

n/a	Involved in the study
☒	☐ Functional and/or effective connectivity
☒	☐ Graph analysis
☒	☐ Multivariate modeling or predictive analysis
